# Exploring the Aggregation Propensity of PHF6 Peptide Segments of the Tau Protein Using Ion Mobility Mass Spectrometry Techniques

**DOI:** 10.1021/acs.analchem.3c04974

**Published:** 2024-03-22

**Authors:** Iuliia Stroganova, Hannah Willenberg, Thaleia Tente, Agathe Depraz Depland, Sjors Bakels, Anouk M. Rijs

**Affiliations:** † Division of Bioanalytical Chemistry, Department of Chemistry and Pharmaceutical Sciences, Amsterdam Institute of Molecular and Life Sciences, Vrije Universiteit Amsterdam, De Boelelaan 1105, Amsterdam 1081 HV, The Netherlands; ‡ Centre for Analytical Sciences Amsterdam, Amsterdam 1098 XH, The Netherlands

## Abstract

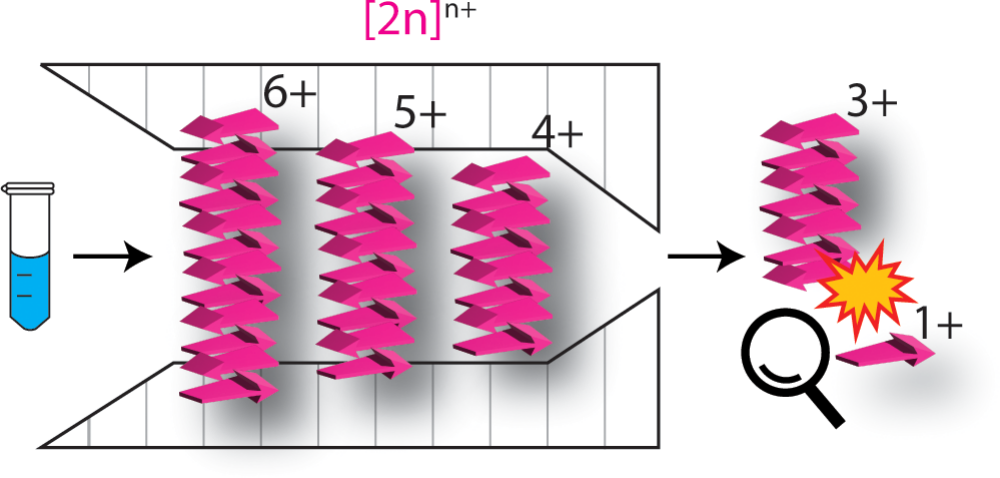

Peptide and protein aggregation involves the formation of oligomeric species, but the complex interplay between oligomers of different conformations and sizes complicates their structural elucidation. Using ion mobility mass spectrometry (IM-MS), we aim to reveal these early steps of aggregation for the Ac-PHF6-NH_2_ peptide segment from tau protein, thereby distinguishing between different oligomeric species and gaining an understanding of the aggregation pathway. An important factor that is often neglected, but which can alter the aggregation propensity of peptides, is the terminal capping groups. Here, we demonstrate the use of IM-MS to probe the early stages of aggregate formation of Ac-PHF6-NH_2_, Ac-PHF6, PHF6-NH_2_, and uncapped PHF6 peptide segments. The aggregation propensity of the four PHF6 segments is confirmed using thioflavin T fluorescence assays and transmission electron microscopy. A novel approach based on post-IM fragmentation and quadrupole selection on the TIMS-Qq-ToF (trapped ion mobility) spectrometer was developed to enhance oligomer assignment, especially for the higher-order aggregates. This approach pushes the limits of IM identification of isobaric species, whose signatures appear closer to each other with increasing oligomer size, and provides new insights into the interpretation of IM-MS data. In addition, TIMS collision cross section values are compared with traveling wave ion mobility (TWIMS) data to evaluate potential instrumental bias in the trapped ion mobility results. The two IM-MS instrumental platforms are based on different ion mobility principles and have different configurations, thereby providing us with valuable insight into the preservation of weakly bound biomolecular complexes such as peptide aggregates.

Aberrant aggregation of peptides and proteins from soluble species into oligomers and ultimately into insoluble, β-sheet-rich amyloid fibrils is a common feature of neurodegenerative diseases (ND).^1^ Although the precise mechanisms of the aggregation process in these NDs remain ambiguous,^2,3^ emerging evidence from several studies indicates that intermediate oligomers have significant cyto- and neurotoxicity.^4,5^ Among the proteins associated with amyloid disorders, the tau protein plays an important role,^6^ particularly in Alzheimer’s disease (AD)^7,8^ and other tauopathies.^9^ Abnormal phosphorylation of tau protein results in its self-aggregation and the formation of neurofibrillary tangles (NFTs). This leads to functional impairment of tau and its dissociation from microtubules and their subsequent destabilization.^10^ A segment that is important in initiating tau protein aggregation has the sequence ^306^VQIVYK^311^. This hexapeptide is also known as PHF6 (Paired Helical Filament 6) and originates from the microtubule-binding domain of the tau protein, specifically from repeat 3 (R3).^11^

In many cases, only a small segment of a protein is essential for the formation of an amyloid core. Such a segment can initiate aggregation and form fibrils by itself.^12,13^ Therefore, these short peptide sequences can be used to elucidate the mechanisms of peptide aggregation,^14,15^ and also for the studies on potential antiamyloidogenic therapeutic agents.^16^ When studying peptide aggregation, it is important to consider how these peptide segments are terminated. For example, N- or C-terminal caps, such as acetyl or amide groups, can remove charge from the peptide termini compared to the uncapped zwitterionic peptide form, thereby controlling terminal interactions,^17^ which results in better mimicking of natural proteins. However, different capping groups can also significantly influence aggregation propensity and fibril morphology.^18^ The effect of capping on aggregation behavior, such as the consequence of peptide charge, electrostatic interactions, and hydrogen bonding,^18−22^ has only been investigated in a limited number of studies, highlighting the need for further investigation of the relationship between peptide capping and aggregation.

Arya et al.^19^ examined the impact of capping on the aggregation propensity and fibril morphology of the PHF6 peptide using thioflavin T (ThT) fluorescence, circular dichroism (CD), and transmission electron microscopy (TEM) techniques along with molecular dynamics simulations. They observed that the uncapped PHF6 peptide in the zwitterionic form with charges at both termini did not show any amyloidogenicity. The PHF6-NH_2_ peptide showed fibril formation only upon addition of heparin, whereas PHF6 peptides with an acetyl group at the N-terminus formed fibrils with different morphologies, even without heparin and independently of the C-terminal capping. This highlights the importance of N-terminal capping, as it increases the aggregation propensity of the peptide. The PHF6 segment has been the subject of several studies using techniques such as Fourier-transform infrared spectroscopy (FTIR), CD, ThT fluorescence, TEM, and X-ray diffraction^23−25^ to shed light on its structural and dynamic properties and aggregation behavior. Although most studies focused on the doubly capped Ac-PHF6-NH_2_ peptide, the PHF6 peptide with different capping groups or the uncapped variant were used in a presumably random manner, demonstrating the lack of a uniform peptide capping protocol when using peptide segments.

Crystallography has shown that, upon fibrillization, the VQIVYK segment forms a steric zipper in which the peptide backbones form a parallel β-sheet,^26^ with the hydrophobic chains of the backbones buried within the zipper structure. This parallel β-sheet arrangement was also demonstrated by solid-state nuclear magnetic resonance experiments.^27^ These structural techniques predominantly provide information on the fully grown fibril, while the abovementioned spectroscopic techniques give structural insights that are averaged over the entire molecular population, which hinders the structural characterization of important, low-abundance, and transient prefibrillar oligomers. Using single-molecule fluorescence resonance energy transfer (smFRET) and kinetic modeling, Kjaergaard et al. showed that for the repeat region of the tau, two major populations with different structures of kinetically stable oligomers were detected;^28^ however, detailed structural information could not be retrieved. Gas-phase infrared action spectroscopy studies focused on the structural properties of the isolated monomeric Ac-PHF6-NHMe peptide and its dimer. Vaden et al. showed that the monomer of Ac-PHF6-NHMe has a β-hairpin-like structure,^29^ while the dimer of this peptide adopts a β-sheet structure in the gas phase; however, they could not distinguish between parallel and antiparallel conformations.^30^ To gain a more detailed understanding of the early stages of peptide aggregation and the intermediate higher order oligomers, we employ ion mobility mass spectrometry (IM-MS) to investigate the aggregation propensity and early-stage aggregation of the PHF6 peptides with different capping groups.

IM-MS is a powerful and sensitive technique that has the capability to separate and characterize complex mixtures and to provide structural information on the overall 3D shape of the analytes.^31,32^ IM-MS has been widely applied to study peptide and protein aggregation, resulting in a molecular-level view of the individual oligomers formed along the aggregation pathway. These studies focused on the differentiation of aggregation pathways,^33^ determination of conformational families of oligomers,^34^ detection of structural transitions during aggregation,^35^ differentiation of inhibitory efficacy of small molecules,^36^ exploration of the structural properties of oligomeric intermediates, and elucidation of their collision cross section values (CCS).^37^ The tau protein and its peptide segments have been studied by various IM-MS techniques, including drift tube ion mobility (DT), traveling wave ion mobility spectrometry (TWIMS), and cyclic ion mobility (cIM), to elucidate the mechanisms of aggregation and to characterize the oligomers and their function.^38−42^ The peptide oligomers are usually noncovalently bound and can be fragmented inside (commercial) IM-MS instruments.^43,44^ One strategy that can be implemented is to optimize instrumental parameters to transfer ions as softly as possible, thereby minimizing fragmentation (ion heating).^45−48^ Another option is to utilize unwanted but often unavoidable fragmentation within the IM-MS instrument. For example, Gray et al. observed the loss of neutral and charged peptides due to post-IM dissociation and correlated this with structural properties and toxicity of weakly bound α-helical oligomers.^49^ Additionally, Borotto and Graham showed how activation prior to IM separation can increase sequence coverage^50^ or allow native collision-induced unfolding (CIU) experiments^51^ without any additional modification of the standard commercial trapped ion mobility instrument (TIMS). In the current work, the soft method^46^ of the TIMS-Qq-TOF for the analysis of transient and fragile oligomers was expanded to a more challenging aggregating system, namely, the tau peptide, which shows a larger and more diverse set of oligomers.

The main focus of this work is to reveal the early stages of aggregation of the PHF6 tau protein segment using IM-MS and therewith investigate the aggregation propensity of PHF6 peptides with different capping groups (Ac-PHF6-NH_2_, Ac-PHF6, PHF6-NH_2_, and PHF6). To support our findings, we used ThT fluorescence and TEM to validate the observed aggregation propensities of the four capped PHF6 peptides. Peptide oligomers are often highly heterogeneous, fragile, and transient in nature, making their separation, identification, and individual characterization challenging. Therefore, we have introduced a novel IM-MS approach based on the ion mobility of fragments generated in the TIMS-Qq-ToF spectrometer, which allows the confident assignment of the intact heterogeneous oligomers without any modeling. To investigate the instrumental bias of the used TIMS-Qq-ToF mass spectrometer for aggregate preservation and/or fragmentation, we compare the results on peptide oligomers of Ac-PHF6-NH_2_ obtained from trapped and traveling wave IM-MS instruments. This allows us to assess whether the experimental approach influences our results, to discuss the advantages of each technique, and to evaluate the interplatform reproducibility of CCS values.

## Experimental Section

### Sample Preparation

The Ac-PHF6-NH_2_, Ac-PHF6, PHF6-NH_2_, and PHF6 peptides (>95% purity) were purchased from Biomatik and used without any further purification. All samples have the following amino acid sequence ^306^VQIVYK^311^ with the N- and C-termini capped via acetylation (Ac−) and/or amidation (−NH_2_), respectively, as indicated in the compound name. Peptide samples were prepared as follows. Approximately 1 mg of peptide was dissolved in 1 mL of 1,3,3,3-hexafluoro-2-propanol (HFIP) and sonicated for 5 min to ensure complete dissolution of the sample. 50 μL of this stock solution was pipetted into aliquots, which were dried in a fume hood for 3–12 h until the HFIP was completely evaporated. The aliquots containing the dried peptide were stored at −20 °C. This aliquot preparation protocol was used for all of the measurements presented in this study. Peptide stock solutions of 50 or 100 μM were prepared in 10 mM ammonium acetate (AA). The peptide solutions were then sonicated for 5 min and further diluted to 50 μM (if necessary) for ion mobility mass spectrometry (IM-MS) experiments. The 10 mM AA solution was prepared by diluting a 5 M stock AA solution (Sigma-Aldrich) in Milli-Q water. The pH of the 10 mM AA solution was adjusted with a 0.5% ammonia:water solution to pH 7.3–7.4. For the experiments presented here on the Ac-PHF6-NH_2_ segment, a fresh solution of 50 μM in 10 mM AA was prepared daily. To investigate the aggregation propensity of the four peptides with different capping groups, each peptide was incubated for 1 day at room temperature at a concentration of 50 μM in 10 mM AA prior to the IM-MS experiments.

### TIMS Operation, Analysis, and Calibration

IM-MS experiments were performed on a TIMS-Qq-ToF (first generation) instrument (Bruker Daltonics GmbH).^46,52−55^ All peptide samples were infused directly via the electrospray ionization (ESI) source at a flow rate of 180 μL/h, operating in the positive mode. IM-MS data were collected over the *m*/*z* 50–3000 range. For all IM-MS measurements, a set of parameters similar to that described previously for peptide oligomers^46^ was used but optimized for the peptide segments studied in this work, to ensure minimal fragmentation of the formed oligomers. All instrumental parameters are summarized in Table S1.

The TIMS cell was filled with nitrogen gas at an inlet pressure of 2.122–2.141 mbar in order to observe oligomeric species within the set mobility range. The main instrumental parameters adapted for this study include capillary voltage, TIMS delta potentials D2, D3, and D6, and ion energy and collision energy (see Figures S1–5 in the Supporting Information). For the quadrupole selection measurements, the data were acquired for 2–10 min depending on the signal intensity of the precursor ion. Inverse reduced mobility (1/*K*_0_, hereinafter referred to as the mobility value) was determined from the peak apex. The ion mobility for each observed oligomer was obtained either by generating an extracted ion mobility spectrum (EIM) from the measured mass spectrum using the full isotopic distribution of a given oligomer in DataAnalysis v5.2 (2019 Bruker Daltonics GmbH) or by using the quadrupole to select the *m*/*z* of interest (±5 *m*/*z*) followed by generating the EIM. The derived CCS values were an average of three measurements of 5 min each obtained on three different days. The CCS values were derived after the calibration procedure in DataAnalysis using the corresponding charge state and the dominant peak in the *m*/*z* peak of the oligomer, and the CCS value was read from the peak apex. Ion mobilities and the CCS values were calibrated as previously described^46^ using Agilent ESI Tuning Mix (*m*/*z* 322, 622, 922, 1222, 1522, 1822, 2122, 2422). The following inverse reduced ion mobility values were used: 0.732, 0.985, 1.190, 1.382, 1.556, 1.729, 1.884, and 2.03 V·s/cm^2^, respectively, for the above *m*/*z* values. Each mass and ion mobility spectrum were calibrated externally in DataAnalysis software after acquisition using the Tuning Mix calibration file measured under identical operating settings.

### Operation and Ion Mobility Calibration for the Photo-Synapt Measurements

The traveling wave ion mobility and mass spectra were measured on the Synapt G2 HDMS (Waters Corp., Manchester, UK), which was modified for spectroscopy in collaboration with MS Vision by adding hexapole pin traps and optical access in front of the time-of-flight section of the instrument (named Photo-Synapt). The data obtained on the Photo-Synapt are referred to as TWIMS experiments below. In the current study, the spectroscopy option was not used, and all the voltages in the hexapole pin traps were optimized for maximum transmission and preservation of the drift time distributions. Freshly prepared peptide samples containing 50 μM of Ac-PHF6-NH_2_ diluted in 10 mM AA (pH 7.3–7.4) were directly infused at a flow rate of 180 μL/h using an ESI source. Mass spectra were acquired over the range *m*/*z* 50–3000 in positive resolution mode. A complete set of instrumental parameters is provided in Table S2. Data acquisition and processing were performed using MassLynx V4.1 software (Waters Corp., Manchester, UK). Exported data were plotted using OriginPro 9.9 software. The details of the CCS calibration procedure can be found in Supporting Information Section S1. Briefly, the logarithm of corrected CCS values and drift times were fitted with a linear regression to determine calibration parameters from the fit. Agilent ESI tuning mix and denatured ubiquitin (30 μM in water/methanol/acetic acid v/v/v 49/49/2) were measured as calibrants with identical parameters as the peptide analyte samples, and their drift tube CCS values measured in N_2_ were used to plot a calibration curve.^56,57^ The drift time distributions extracted from the *m*/*z* values of oligomers were smoothed in MassLynx software using a Savitzky–Golay filter, and the drift time values were read from the peak apex. The CCS values of the Ac-PHF6-NH_2_ peptide reported here are the averages of three measurements.

### Thioflavin T Fluorescence Assay

The protocol for ThT assays and fibril visualization by TEM was adapted from a study by Arya et al.^19^ Peptide solutions were diluted with 10 mM AA (pH 7.3–7.4) or 10 mM AA solution enriched with either 150 mM NaCl or 1.15 μM heparin (Sigma-Aldrich, H4784, the molar concentration is calculated based on the average molecular weight of 15 kDa) and sonicated for 5 min. The 20 mM ThT stock solution was dissolved in Milli-Q and filtered through a 0.2 μm syringe-driven filter unit (Millex). 10 μL of the 500 μM ThT solution was added to a black 384-well plate with a clear bottom (Thermo Scientific, Cat. No. 242764). Subsequently, 90 μL of peptide samples was added to the wells, resulting in a final concentration of 150 μM peptide and 50 μM ThT in a well. The plate was then sealed with an optical adhesive film (Applied Biosystems, Cat. No. 4360954). The ThT fluorescence assays were performed on a Varioskan LUX plate reader (Thermo Scientific, SN LL163801). The excitation and emission wavelengths were set at 440 and 485 nm, respectively, with a bandwidth of 5 nm. All readings were performed at 37 °C without shaking between measurements. The first time point corresponds to the measurements that lasted for 22 h, and readings were done every 5 min. The well plate with peptides was then kept at 37 °C in an incubator. For later time points, the measurements were made every 5 min four times, but the first reading was not included because the intensity was much lower. All measurements were performed in triplicate (three wells), and the fluorescence was measured from the top of the wells.

### TEM

Peptide solutions of 150 μM were diluted with 10 mM AA (pH 7.3–7.4) or 10 mM AA solution enriched with either 150 mM NaCl or 1.15 μM heparin and sonicated for 3 min. Peptide samples were incubated at 37 °C for 9 days without agitation prior to TEM imaging. Peptide solutions were then spotted on freshly glow-discharged carbon/Formvar-coated mesh grids. After blotting off the excess liquid, the samples were contrasted by 2% uranylacetate (Polysciences Inc., Cat No 21447-25) in water for 1 min, the excess stain was blotted off, and grids were air-dried. Fibrillar structures were imaged on an 80 kV Tecnai 12 (Thermo Fisher) TEM at 135k and 300k magnification using a 2k × 2k pixel CCD side-mounted camera (Veleta, EMSIS GmbH).

## Results and Discussion

### Oligomers of the Ac-PHF6-NH_2_ Peptide: Observations from IM-MS Experiments

**Figure 1:**
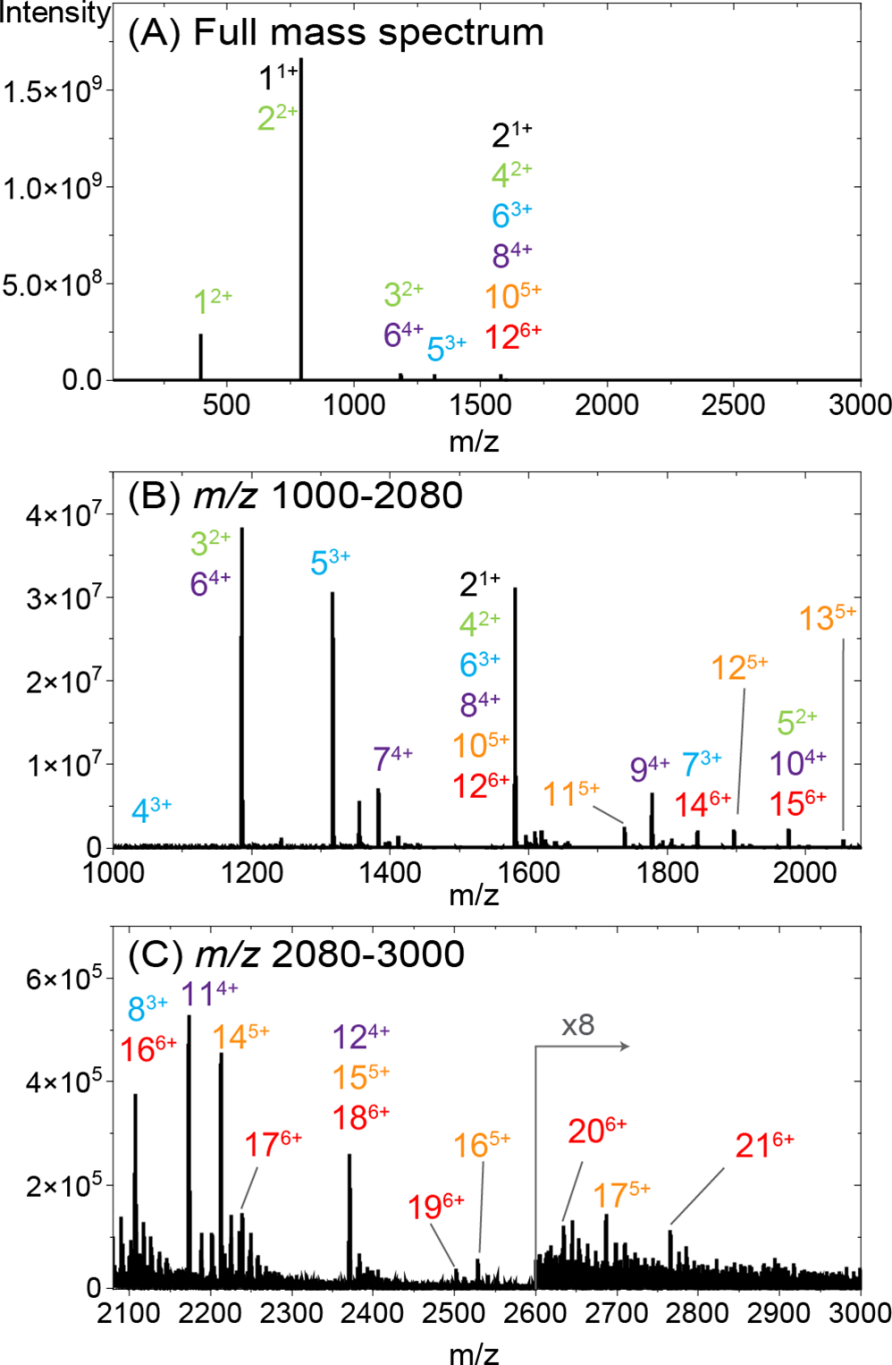
(A) Mass spectrum of the Ac-PHF6-NH_2_ peptide in the *m*/*z* 50–3000 region (50 μM freshly prepared solution in 10 mM ammonium acetate solution); *n*^z+^ notation represents oligomers, where *n* indicates the oligomer number and *z* the charge state. (B, C) A zoomed-in view of the higher *m*/*z* regions, highlighting the present higher-order oligomers.

The mass spectrum of the freshly prepared Ac-PHF6-NH_2_ peptide shows a wide distribution of multiply charged oligomeric species ranging from singly charged monomers to 21^6+^ (Figure 1). The oligomers are labeled as *n*^z+^ ([*nM*+*zH*]^z+^), where *n* is the number of monomer units in the oligomer and *z* is the number of charges. The assignment is made by analyzing mass (isotopic distribution) and ion mobility spectra, which will be discussed in detail below. Under the investigated conditions and concentrations, no significant changes were observed in the mass and ion mobility spectra over time. Using a ThT fluorescence assay, in which a small dye ThT fluoresces when it binds to β-sheet fibrils,^58^ we show that the aggregation of Ac-PHF6-NH_2_ and the lag phase are rather slow (about 7–9 days) for the peptide concentration and AA solution used for this study. Aggregation and fibril formation are significantly faster when higher concentrations of AA are used or when heparin or NaCl salt is added to the solution (see Figures S6 and S7). The former results from an increase in ThT binding affinity and fluorescence intensity with increasing ionic strength,^59^ while the addition of anionic factors such as heparin is known to accelerate tau aggregation.^11^ Fibril formation for the Ac-PHF6-NH_2_ peptide after incubation at 37 °C for 9 days was confirmed by TEM (see Figures S8 and S9 for more details including a comparison with the data from^19^). The fibrils of Ac-PHF6-NH_2_ have a characteristic twisted morphology. Therefore, we can conclude that the aggregation of the Ac-PHF6-NH_2_ peptide is slow under our MS conditions, which allows us to study the soluble oligomers formed at the early time points of the aggregation process.

**Figure 2:**
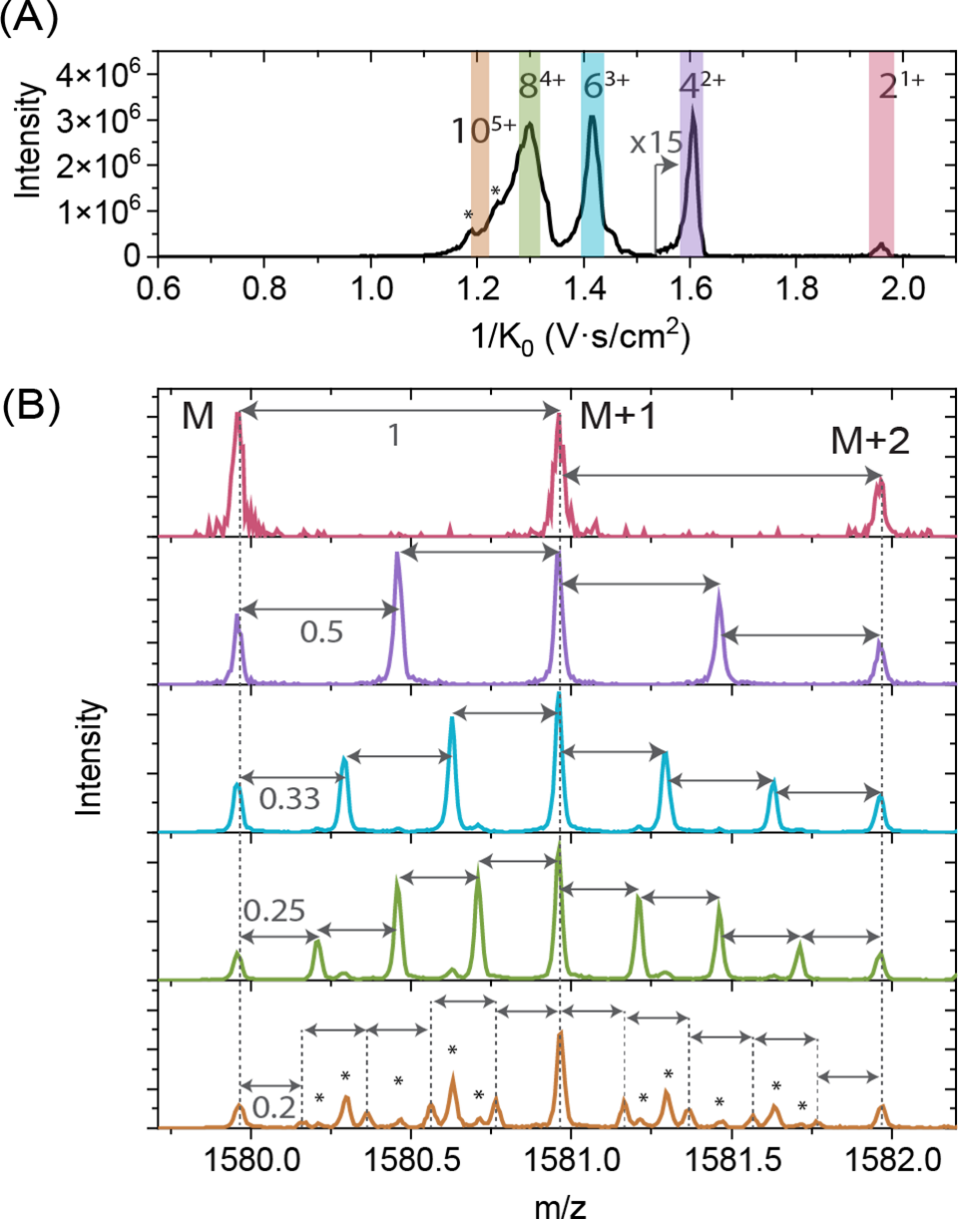
(A) Extracted ion mobility spectrum of *m*/*z* 1580 of the Ac-PHF6-NH_2_ peptide; highlighted peaks correspond to the different oligomers. The region above 1.535 V·s/cm^2^ has been scaled, as indicated for clarity. The asterisks correspond to the fragments into *m*/*z* 1580. (B) Extracted mass spectra from the mobility peaks shown in (A) zoomed-in to the *m*/*z* 1580 region. The distance between the peaks indicates the charge state of the oligomers, i.e., the pink trace is singly charged dimer 2^1+^, purple is 4^2+^, blue is 6^3+^, green is 8^4+^, and the region in orange corresponds to 10^5+^. The asterisks here show the peaks corresponding to the lower charge states oligomers fragmented into 10^5+^.

IM-MS allows the separation and identification of individual oligomers with the same *m*/*z* ratio. In Figure 2, we focus on the *m*/*z* 1580 peak originating from the singly protonated dimer (2^1+^) and higher-order oligomers with the same [2*n*]^*n*z+^ oligomer to charge ratio. Under our soft experimental conditions, this is the most abundant peak in MS with multiple oligomeric species present. Note that when the experimental conditions are harsher, oligomer fragmentation is observed, resulting in the disappearance of the higher order oligomers in this *m*/*z* channel (see Figures S1–S4). The procedure for identifying the oligomeric species, leading to the peak assignment shown in Figure 1, is described below for the *m*/*z* 1580 peak. The extracted ion mobility spectra of the other *m/*z peaks observed in the mass spectrum reported in Figure 1 and their peak assignments are shown in Section S2 of Supporting Information.

Figure 2A shows the extracted ion mobility spectrum of *m*/*z* 1580. Four major peaks are observed, which are highlighted in pink, purple, blue, and green, and a shoulder at lower reduced ion mobilities is shown in orange. For each of the mobility peaks, a mass spectrum was extracted, and the isotopic distribution was evaluated to determine the oligomer charge state and size (see Figure 2B). For example, the peak at 1/*K*_0_ = 1.959 V·s/cm^2^ (pink) corresponds to the singly charged dimer 2^1+^, the peak at 1/*K*_0_ = 1.606 V·s/cm^2^ (purple) originates from the doubly charged tetramer 4^2+^, the peak at 1/*K*_0_ = 1.416 V·s/cm^2^ (blue) corresponds to the triply charged hexamer 6^3+^, and the peak at 1/*K*_0_ = 1.299 V·s/cm^2^ (green) is the quadruply charged octamer 8^4+^. This octamer peak at 1.299 V·s/cm^2^ has a shoulder to the left, indicating the possible presence of even higher-order oligomers. The two peaks marked with an asterisk in this shoulder are not originating from the higher order oligomers of [2*n*]^*n*z+^ but are the result of fragmentation into the *m*/*z* 1580 channel after the TIMS cell (see Section S2, Figure D4). The extracted mass spectrum from the ion mobility of 1/*K*_0_ = 1.203 ± 0.016 V·s/cm^2^ (orange trace in Figure 2B) represents a combination of multiple isotopic distributions, among which a set of low-intensity peaks associated with 5+ charged species could be identified. This indicates the presence of 10^5+^ species, but there is no clear ion mobility peak associated with this oligomer. In addition, smaller oligomers with 2+, 3+, and 4+ charge states appear in this *m*/*z* channel of the extracted mass spectrum due to their fragmentation into 10^5+^ (marked with asterisks on the orange trace in Figure 2B). The presence of higher oligomers (>10 monomers) could not be derived unambiguously using this analysis workflow.

### Quadrupole Selection Experiments: Peak Assignment in IM-MS

**Figure 3:**
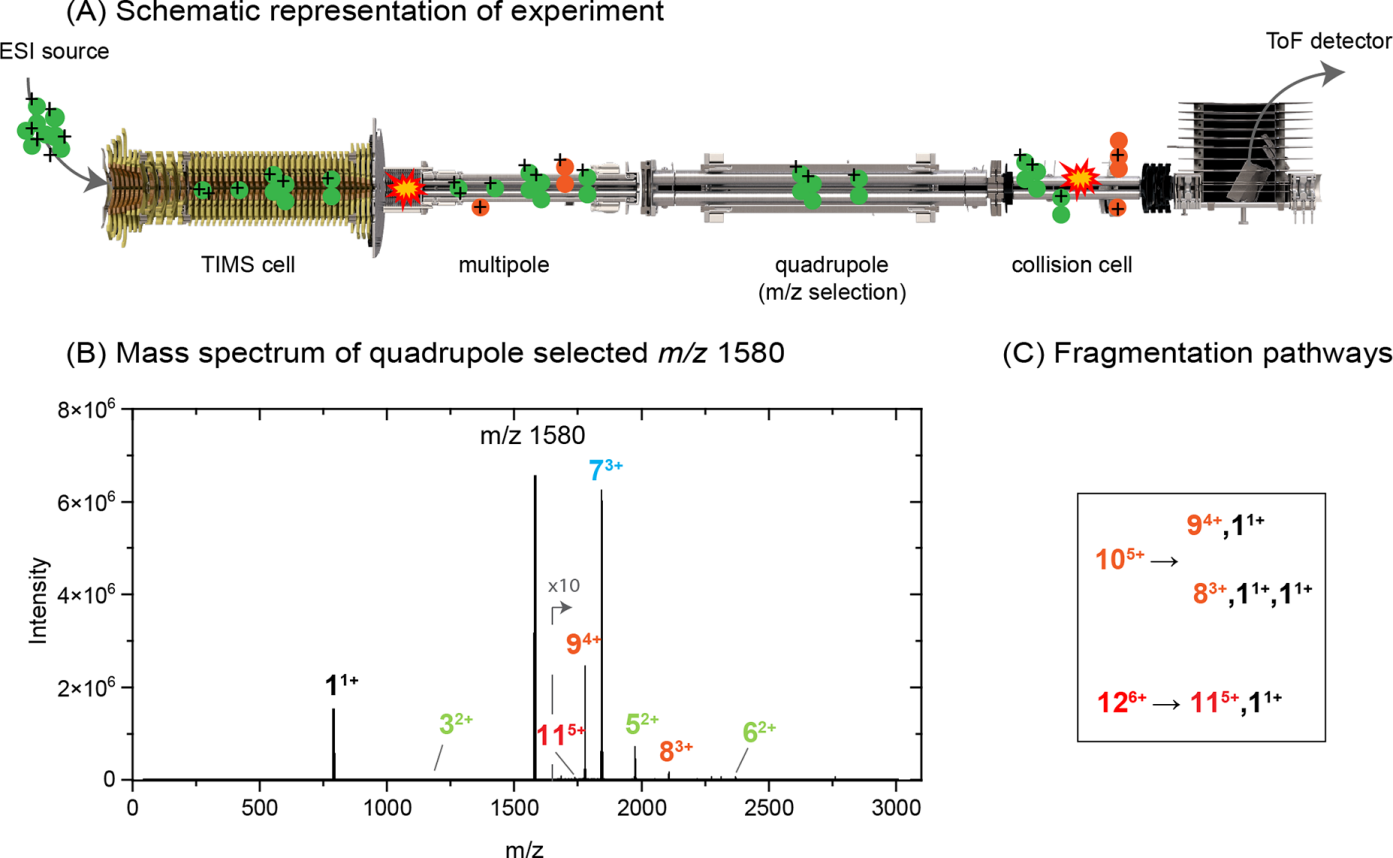
(A) Schematic representation of the quadrupole selection experiments. Oligomers with the same *m*/*z* ratio [2*n*]^*n*z+^ are selected in the quadrupole (in green), while the orange ions represent fragment ions that could appear in two regions in the TIMS-Qq-ToF (highlighted in red). (B) Mass spectrum of the quadrupole-selected ions with *m*/*z* 1580 of the Ac-PHF6-NH_2_ peptide. The region above *m*/*z* 1650 has been multiplied by 10 to show the fragment ions more clearly. (C) Fragmentation pathways of higher-order oligomers with *m*/*z* 1580, resulting in the observed fragment ions.

In order to assign all the features observed in the ion mobility spectra of *m*/*z* 1580, such as the origin of the tail at 1/*K*_0_ = 1.015–1.263 V·s/cm^2^, we use the quadrupole of the TIMS-Qq-ToF instrument to filter the *m*/*z* value of interest. A schematic overview of this experiment is shown in Figure 3A. As discussed in our previous study,^46^ even under our soft conditions, the relatively fragile oligomers can fragment at various stages as they pass through the spectrometer (indicated by the orange dots in Figure 3A). This occurs mainly at the TIMS-multipole interface and in the collision cell.^43^ To confirm our assignment and to identify the origin of the remaining signatures observed in the ion mobility spectra, the quadrupole filtering experiment was repeated for all Ac-PHF6-NH_2_ oligomers with sufficient signal intensity to be selected by the quadrupole (assignments can be found in Section S2). Here, we will focus on the quadrupole-selected oligomers with *m*/*z* 1580 ([2*n*]^*n*z+^) of the Ac-PHF6-NH_2_ peptide.

**Figure 4:**
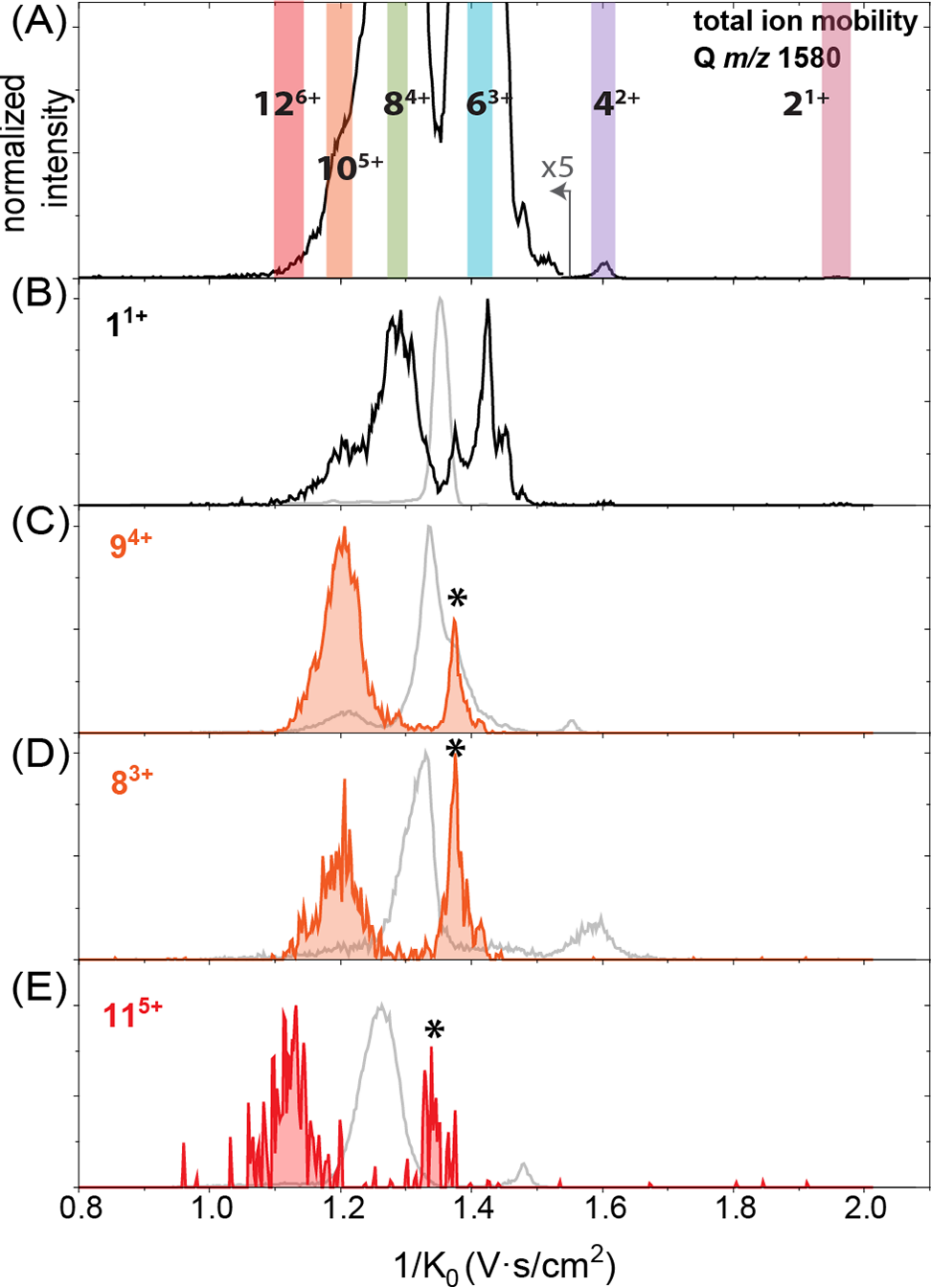
(A) Total ion mobility spectrum of the quadrupole-selected *m*/*z* 1580 of the Ac-PHF6-NH_2_ peptide. The region below 1/K_0_ 1.54 V·s/cm^2^ was multiplied by 5. (B–E) Extracted ion mobility spectra of the fragment ions (1^1+^, 9^4+^, 8^3+^, and 11^5+^) formed in the collision cell from [2*n*]^*n*z+^ oligomers. The gray traces represent the extracted ion mobilities of the corresponding intact oligomers (*m*/*z* 790.5, 1777, 2106, and 1738) when measured without quadrupole selection.

Figure 3B shows the mass spectrum with the quadrupole mass filter set at *m*/*z* 1580. The most intense peak corresponds to the precursor ion; however, several other peaks are also present. These peaks result from the fragmentation of the quadrupole-selected oligomers with *m*/*z* 1580 in the collision cell. Figure 3C shows possible fragmentation pathways of these fragment ions that can be formed from the higher-order oligomers with *m*/*z* 1580, i.e., 10^5+^ and 12^6+^. Figure 4A shows the total ion mobility spectra when *m*/*z* 1580 is selected with the quadrupole. To assign there the region of [2*n*]^*n*z+^ higher-order oligomers (1/*K*_0_ = 1.0–1.2 V·s/cm^2^), we derived the mobility spectra of the fragment ions indicated in Figure 3C. The quadrupole filtered, extracted ion mobilities of the fragment ions of the 10^5+^ oligomer, namely, 1^1+^, 8^3+^, and 9^4+^, are shown in Figure 4B–D. The gray lines represent the extracted ion mobilities of *m*/*z* 790.5, 2106, and 1777 of the intact 1^1+^, 8^3+^, and 9^4+^ oligomers, respectively. Figure 4B–E clearly shows that the mobility spectra of the intact oligomers (light gray) are different from the mobility spectra of the fragments (black, orange, and red). The 1^1+^, 8^3+^, and 9^4+^ fragment ions (from the 10^5+^ oligomer) all show a mobility peak at 1/*K*_0_ = 1.203 V·s/cm^2^, which coincides with the assumed position of the 10^5+^ oligomer, as shown in Figure 2A. The same analysis was carried out for the 12^6+^ oligomer with fragmentation resulting in the 1^1+^ and 11^5+^ fragment ions. The 11^5+^ fragment (red trace, Figure 4E) has a distinct peak at 1/*K*_0_ = 1.126 V·s/cm^2^ in the extracted ion mobility spectrum, which coincides with the small shoulder of the 1^1+^ fragment, indicating the position of the 12^6+^ oligomer. Therefore, the peaks at 1/*K*_0_ = 1.203 and 1.126 V·s/cm^2^ are assigned to the 10^5+^ and 12^6+^ oligomers, respectively. The ion mobility spectra of the other fragment ions from *m*/*z* 1580 are shown in Section S2, Figure D4. The additional ion mobility peaks of the fragment ions (marked with asterisks in Figure 4C–E) originate from the fragmentation of other larger oligomers into *m/*z 1580 at the TIMS-multipole interface, mainly due to the neutral loss of the monomer^49^ and their subsequent dissociation into 8^3+^, 9^4+^, and 11^5+^ in the collision cell. For example, an intact oligomer of 13^5+^ fragments at the TIMS-multipole interface into 10^5+^, which passes through the quadrupole and further dissociates into 8^3+^ or 9^4+^ in the collision cell. The IMS signature of the 13^5+^ is therefore preserved and appears as additional peaks at a higher 1/*K*_0_ of the fragment ions (marked with asterisks). The detailed assignment of these peaks is shown in Figure S10.

Even with our optimized soft conditions for the TIMS-Qq-ToF,^43,46^ fragmentation still occurs at the TIMS-multipole interface and between the quadrupole and the ToF (collision cell region). The latter can be clearly seen in Figure 4C, where the extracted ion mobility of the intact 9^4+^ oligomer (gray) shows an additional peak at 1.2 V·s/cm^2^, resulting from the fragmentation of the 10^5+^ oligomeric species into the *m*/*z* channel of 9^4+^ in the collision cell (see Figure Q2 in Section S2). Fragmentation at both the TIMS-multipole interface and the collision cell therefore results in spurious peaks in the ion mobility spectra. Here, we show that by recording ion mobility spectra with and without quadrupole selection, a large number of oligomers resulting from the aggregation of the Ac-PHF6-NH_2_ peptide segment of the tau protein can be identified by their *m*/*z* and mobility values. This approach is of great value when the ion mobility and the corresponding extracted mass spectra are inconclusive and complicated by fragmentation. When used on the high-resolution IM-MS platform, the assignment of higher-order isobaric oligomers is enhanced, the ion mobility signatures of which become more closely spaced as the oligomers increase in size.

### Comparison of TIMS and TWIMS Mass Spectrometry Ion Mobility Data for Ac-PHF6-NH_2_ Oligomers

**Figure 5:**
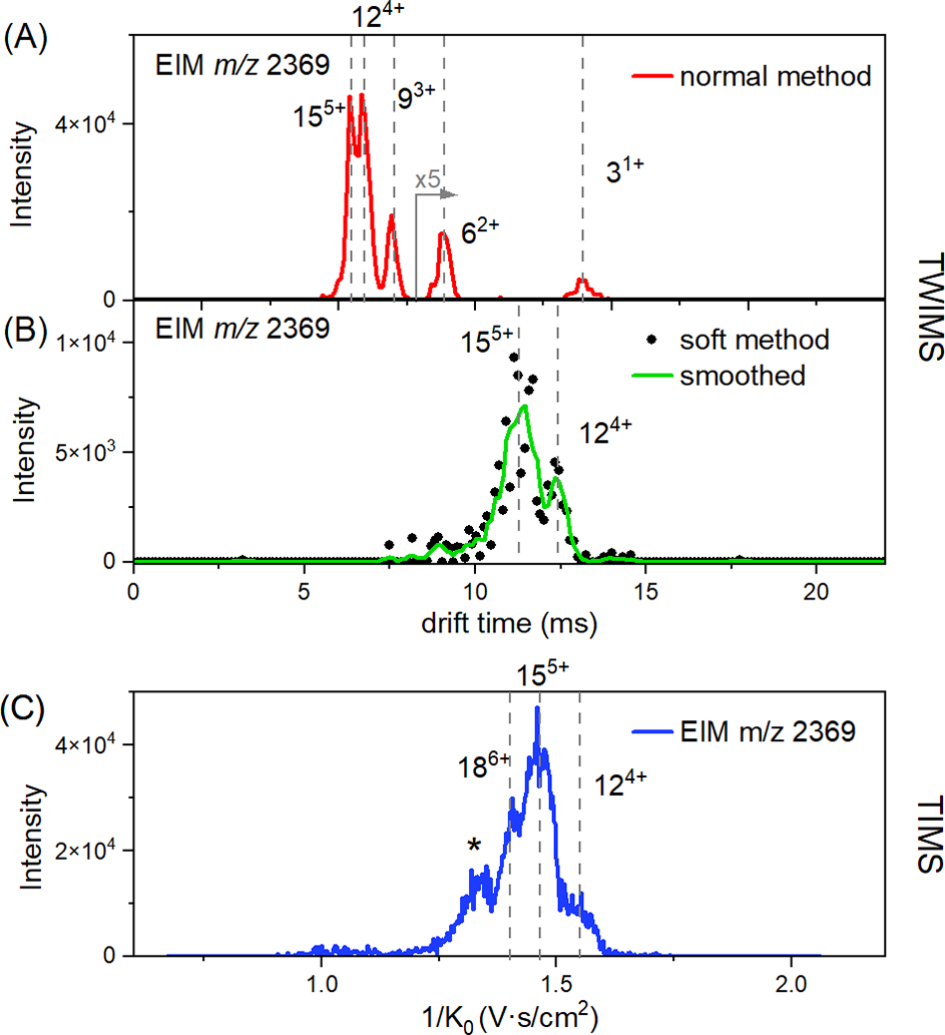
(A) Extracted ion mobility spectrum of *m*/*z* 2369 of the Ac-PHF6-NH_2_ peptide measured on the TWIMS instrument, highlighting the peak assignments corresponding to [3*n*]^*n*z+^ oligomers. The region above 8 ms has been scaled as indicated for clarity. (B) Extracted ion mobility spectrum of *m*/*z* 2369 of the Ac-PHF6-NH_2_ peptide measured on the TWIMS instrument with softer settings. The green trace corresponds to the raw data smoothed with the Savitzky–Golay filter in MassLynx. (C) Extracted ion mobility spectrum of *m*/*z* 2369 of the Ac-PHF6-NH_2_ peptide measured on the TIMS instrument. The peak marked with the asterisks could not be assigned to the higher-order oligomer of [3*n*]^*n*z+^, but it originates from fragmentation after the ion mobility cell.

To investigate to what extent the number and type of observed oligomers are influenced by the employed TIMS-Qq-ToF spectrometer, we have repeated the measurements of the Ac-PHF6-NH_2_ segment using the Photo-Synapt (TWIMS) ion mobility mass spectrometer. In general, the same oligomers (in size and number) were observed for both instruments. Figure 5 shows the extracted ion mobility spectra of *m*/*z* 2369 corresponding to [3*n*]^*n*z+^ oligomers. Depending on the settings of the Photo-Synapt, the ion mobility spectra show different abundances of the oligomers, in agreement with the TIMS experiments (Figures S1–S4). Under normal operating conditions, the presence of lower charge state oligomers can be seen (Figure 5A) but with softer settings (lower source voltages and ion mobility parameters) the abundance of higher charge state oligomers increases (Figure 5B). At the same time, softer TWIMS settings allowed other oligomers (e.g., 11^5+^ and 12^5+^) to be detected in the mass spectrum (see Figure S11). The downside of the softer settings is reduced ion signal intensity, especially for the lower-intensity ions (*m*/*z* > 2500). To improve this, we have recorded data over a longer period of time (20 min). An overview of the Photo-Synapt settings can be found in Table S2.

The extracted ion mobility spectrum shown in Figure 5B (TWIMS) has a peak pattern similar to that observed with TIMS (Figure 5C). However, the presence of 18^6+^ oligomers could not be confirmed on the TWIMS instrument due to the low signal and slightly lower MS resolution, resulting in an inconclusive isotopic pattern. The Photo-Synapt allowed us to measure the ion mobility of a wider range of oligomers with the same *m*/*z* using a single method (Figure 5A), whereas with the TIMS, due to the scanning mode of operation, the mobility window is defined by the voltages on the electric field gradient.^31^ However, the TIMS instrument has a higher IM-resolving power, which allows for the separation of higher-order oligomers. It should be noted that the methods on both instruments have been optimized for a wide range of both *m*/*z* and ion mobility values in order to capture as many oligomers as possible with one method. Therefore, the optimal settings for a particular oligomer may differ from those shown here. For further comparison with the TIMS instrument, the data obtained with softer settings of Photo-Synapt are used.

To correlate the peak assignments on the TWIMS- and TIMS-based spectrometers, we provide a comparison of the calibrated CCS values of all of the coinciding oligomers measured on both instruments. A summary of the averaged ^TIM^CCS_N2_ (TIMS) and ^TW^CCS_N2_ (TWIMS) values, their standard deviations, and the relative differences between the two instruments are shown in Table S3. The average relative error for all oligomers found on both instruments is 1.7%, with the highest error being 4.6%. The comparative studies between ^DT^CCS_N2_ and ^TW^CCS_N2_ values showed ΔCCS deviation between the methods of about 1–2%,^60,61^ while the CCS biases within 1% for TIMS and within 2% for TWIMS with respect to DT values were observed for steroids.^62^ Regueiro et al. compared ^TW^CCS_N2_ values of pesticides with ^DT^CCS_N2_ values from the literature and found the difference of up to ±2.3%.^63^ Therefore, based on the literature reports, there is a generally accepted variation of about 2% for the reproducibility of the CCS values.^63,64^ We also noted that our ^TIM^CCS_N2_ values are usually smaller than the ^TW^CCS_N2_ values, which could be due to the external calibration procedures and the use of different calibrants for two methods that potentially causes the observed bias.^61,62^ The data from both instruments show an excellent linear correlation between each other when fitted with a linear relationship with *R*^2^ > 0.999 (see Figure S12), which again confirms that the ^TIM^CCS_N2_ and ^TW^CCS_N2_ are similar. Since the same oligomers were observed by both ion mobility methods and they have similar CCS values (indicating similar structures), we can conclude that there is no significant instrumental bias. The observed species reflect the early stage oligomers present in the Ac-PHF6-NH_2_ sample.

### Effect of Terminal Capping on Aggregation Propensity: TIMS Data

**Figure 6:**
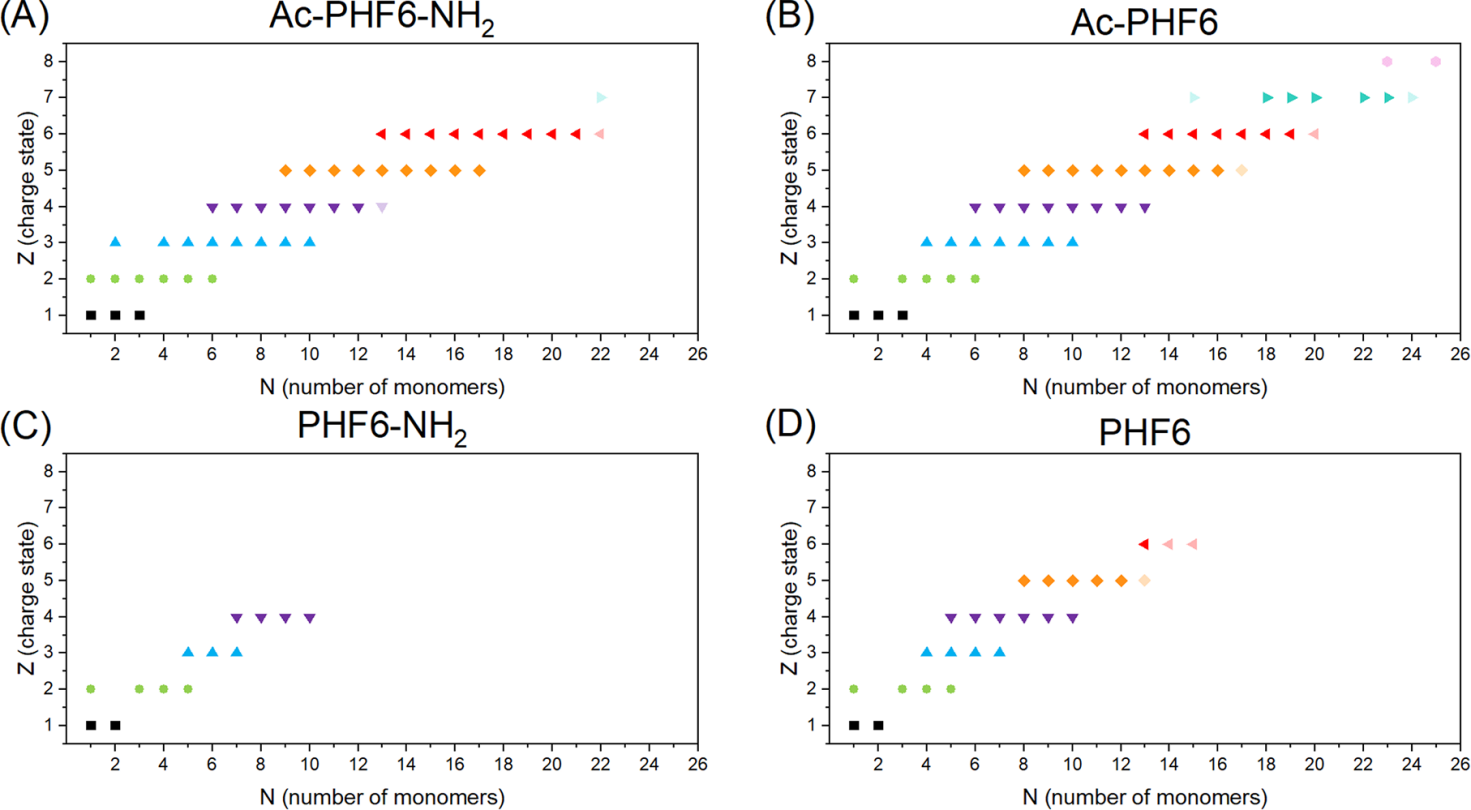
Summary of oligomer abundances of four differently capped peptides Ac-PHF6-NH_2_ (A), Ac-PHF6 (B), PHF6-NH_2_ (C), and PHF6 (D). Every point here represents a found oligomer with a charge state *Z* and a number of monomers *N*. The faded points correspond to the oligomers that are at the noise level.

To evaluate the effect of terminal capping on the aggregation propensity of PHF6 peptides, we measured all four peptides, incubated for 24 h prior to the experiment, on the same day using the TIMS instrument. To ensure that the signal did not change between measurements, the intensity of the Tuning Mix was monitored throughout the day. The detected oligomers shown here are those that were found in the mass spectrum with a signal-to-noise ratio (S/N) greater than 3. Figure 6 shows that the Ac-PHF6-NH_2_ and Ac-PHF6 segments have much more oligomers than PHF6-NH_2_ and PHF6, which is in agreement with the work of Arya et al.^19^ They showed in simulations that uncapped N-terminal PHF6 peptides could not form large oligomers (>7 monomers). This is in line with our experiments where we also observed a limited oligomer size for the uncapped N-terminus segments, although a slightly larger oligomer size was found.

The PHF6 peptides with an acetyl-capped N-terminus form higher-order oligomers with more than 20 monomer units and up to 8+ charge states, whereas for PHF6 and PHF6-NH_2_, the maximum oligomer size remains at about 10 monomers. This could imply that when about 10 monomer units are formed, a junction on the aggregation pathway is reached, where the oligomers either continue to grow and transform into larger β-sheet structures toward fibrils or their growth stagnates. A similar observation was made by Li et al., who used Monte Carlo simulations to study the early stages of oligomerization of the Ac-PHF6-NH_2_ peptide.^65^ These simulations revealed a two-step behavior. First, during the nucleation step, many metastable structures are formed before the appearance of stable aggregates, which have β-sheet content and consist of at least 10 monomers. However, for these oligomers to grow further into a fibril, they need to undergo an antiparallel to parallel structural rearrangement, which can be a time-limiting step. Larger aggregates with 16–20 monomeric units of Ac-PHF6-NH_2_ were observed during oligomer growth in their simulations, suggesting that the higher-order oligomers that we observed for Ac-PHF6-NH_2_ and Ac-PHF6 of 22–25 monomers can potentially be the on-pathway aggregates. The aggregation propensity of the four peptides is confirmed by our ThT assays and TEM data (see Figures S6 and S9), showing that only Ac-PHF6-NH_2_ and Ac-PHF6 segments were able to form fibrils in solution. Under our low salt and MS compatible conditions, fibril formation for Ac-PHF6-NH_2_ and Ac-PHF6 peptides occurred only after 7–9 days, which may indicate that the higher-order oligomers need to undergo structural rearrangement before they can lead to the formation of fully grown fibrils. However, based on our set of experiments, we cannot say whether the oligomers we observed have parallel or antiparallel β-sheet content. A follow-up study will be performed using IR action spectroscopy on our Photo-Synapt instrument to obtain structural information on the β-sheet signatures of the oligomers.

## Conclusions

The aggregation properties of the PHF6 peptide segments of the tau protein were investigated by using two ion mobility mass spectrometry techniques. By carefully tuning the instrumental settings of the TIMS-Qq-ToF instrument, the early-stage oligomers of the Ac-PHF6-NH_2_ peptide up to 21 monomers were separated and identified. This soft method can still lead to the dissociation of oligomers before they reach the detector; however, we utilized post-IM fragmentation and quadrupole selection on the TIMS-Qq-ToF instrument, which allowed us to improve the identification of oligomers. The ion mobility spectra of the fragment ions were used to assign peaks in the extracted ion mobility of *m*/*z* 1580 that would otherwise be hidden without quadrupole filtering. This approach provides valuable insight for studies of complex heterogeneous mixtures of weakly bound species that are easily fragmented inside commercial IM-MS instruments. It can be performed on any ion mobility mass spectrometer, although the physical sequence of the ion mobility cell, quadrupole, and collision cell may vary depending on the manufacturer, which should be considered when analyzing the data.

To check for instrumental bias in the TIMS results and to verify that the oligomers are solution-derived rather than instrument-generated, we compared the ion mobility data of the Ac-PHF6-NH_2_ oligomers from TIMS and TWIMS instruments. Two IM-MS platforms based on different physical principles gave similar results; however, the instrumental parameters still need to be carefully tuned as they can affect the relative abundances of the ions. The ^TIM^CCS_N2_ and ^TW^CCS_N2_ values had an average relative difference of about 1.7%, which is within the currently accepted range. Overall, this confirms our ion mobility assignments and proves that the observed gas phase oligomer conformations are not instrumentally biased.

Finally, we demonstrated how the terminal capping of the PHF6 peptides affects their aggregation propensity. Our MS data from the TIMS-Qq-ToF showed that the Ac-PHF6-NH_2_ and Ac-PHF6 peptides formed much more oligomers, indicating a higher aggregation propensity than PHF6-NH_2_ and PHF6, in agreement with ThT fluorescence assays and TEM results. The relatively slow aggregation of Ac-PHF6-NH_2_ and Ac-PHF6 peptides under our MS compatible conditions may be related to the structural reorganization that needs to happen before the mature fibrils can be formed,^14^ which can be accelerated by heparin^11^ or higher buffer concentrations. Although it was shown in the literature that the PHF6 peptide forms layered β-sheet oligomeric structures,^66^ we cannot show this with our current data. By probing aggregation happening in solution in three different states of matter, gas phase (IM-MS), liquid (ThT assays), and solid (TEM), we obtained a coherent and consistent picture of the aggregation propensity of the PHF6 peptides with different capping groups.

## Data Availability

The data underlying this study are openly available in DataCite Commons at https://doi.org/10.48338/vu01-nh2axm.

## Supporting Information Available

Instrumental parameters of TIMS and TWIMS, effect of the instrumental settings on the oligomer abundancies, details of the CCS calibration procedure for the Photo-Synapt, ThT assays and TEM data for PHF6 peptides, comparison of the CCS values of the Ac-PHF6-NH_2_ peptide measured with TIMS and TWIMS, and details of the ion mobility peak assignments of peaks on TIMS (PDF)
