# Supplementary material for: Exploring the Aggregation Propensity of PHF6 Peptide Segments of the Tau Protein Using Ion Mobility Mass Spectrometry Techniques
Source: Anal Chem. 2024 Mar 22;96(13):5115–24. doi: 10.1021/acs.analchem.3c04974 (PMC10993201; doi:10.1021/acs.analchem.3c04974)
Supplement: Supplementary file 1 — ac3c04974_si_001.pdf [file ac3c04974_si_001.pdf]

Supporting Information

Exploring the Aggregation Propensity of PHF6 Peptide Segments of the Tau Protein using Ion Mobility Mass Spectrometry Techniques

Iuliia Stroganova[1,2], Hannah Willenberg[1,†], Thaleia Tente[1], Agathe Depraz Depland[1,2], Sjors Bakels[1,2], and Anouk M. Rijs[1,2*]

1. Division of Bioanalytical Chemistry, Department of Chemistry and Pharmaceutical Sciences, Amsterdam Institute of Molecular and Life Sciences, Vrije Universiteit Amsterdam, De Boelelaan 1105, 1081 HV Amsterdam, the Netherlands

2. Centre for Analytical Sciences Amsterdam, 1098 XH, Amsterdam, the Netherlands

†Institute for Molecules and Materials, Radboud University Nijmegen, Toernooiveld 1, Nijmegen, 6525 ED, The Netherlands.

Correspondence to: Anouk M. Rijs, e-mail: a.m.rijs@vu.nl

Contents

Table S1. Instrumental parameters on TIMS for IM-MS measurements............S2

Figure S1. Effect of capillary voltage in the TIMS-Qq-ToF on oligomer abundances............S3

Figure S2. Effect of D2 potential in the TIMS-Qq-ToF on the oligomer abundances S3

Figure S3. Effect of D3 potential in the TIMS-Qq-ToF on oligomer abundances S4

Figure S4. Effect of D6 potential in the TIMS-Qq-ToF on oligomer abundances S4

Figure S5. Effect of ion energy and collision energy on fragmentation in the collision cell............S5

Table S2. Instrumental parameters on The Photo-Synapt for TWIMS IM-MS measurements............S5

Section S1. CCS Calibration Procedure for Photo-Synapt (TWIMS) Data............S7

Figures S6-S7. ThT fluorescence assays for PHF6 peptides S8

Figure S8. TEM images of Ac-PHF6-NH_2 peptide S9

Figure S9. TEM images of PHF6 peptides S10

Figure S10. Ion mobility assignment of fragment ions due to neutral loss of monomer............S11

Figure S11. Comparison of mass spectra with normal and soft settings on the Photo-Synapt............S12

Table S3 and Figure S12. Comparison of CCS values of Ac-PHF6-NH_2 peptide oligomers obtained by TIMS and TWIMS ion mobility techniques S13

Section S2. Ion Mobility Peak Assignments of the TIMS data S15

References............S26

Table S1. Instrumental parameters on TIMS for IM-MS measurements.

**Source**	
**Capillary voltage**	*2500 V*
**End Plate offset**	*200 V*
**Nebulizer pressure**	*0.8 Bar*
**Dry gas flow**	*4 L/min*
**Dry temperature**	*80°C*
**General parameters**	
**Funnel 2 RF**	*300 Vpp*
**Multipole RF**	*400 Vpp*
**isCID energy**	*0 eV*
**Deflection delta**	*10 V*
**Low mass**	*300 m/z*
**Ion energy**	*9 eV*
**Collision energy**	*0 eV*
**Collision RF**	*1300 Vpp*
**Transfer time**	*90 µs*
**Pre Pulse storage**	*14 µs*
**IMS parameters**	
**D1**	*10 V*
**D2**	*0 V*
**D3**	*20 V*
**D4**	*20 V*
**D5**	*10 V*
**D6**	*10 V*
**Funnel 1 RF**	*250 Vpp*
**Collision Cell In**	*200 V*
**Accumulation time**	*5 ms*
**Ramp time**	*100 ms*

Figure S1. Effect of capillary voltage in the TIMS-Qq-ToF on oligomer abundances

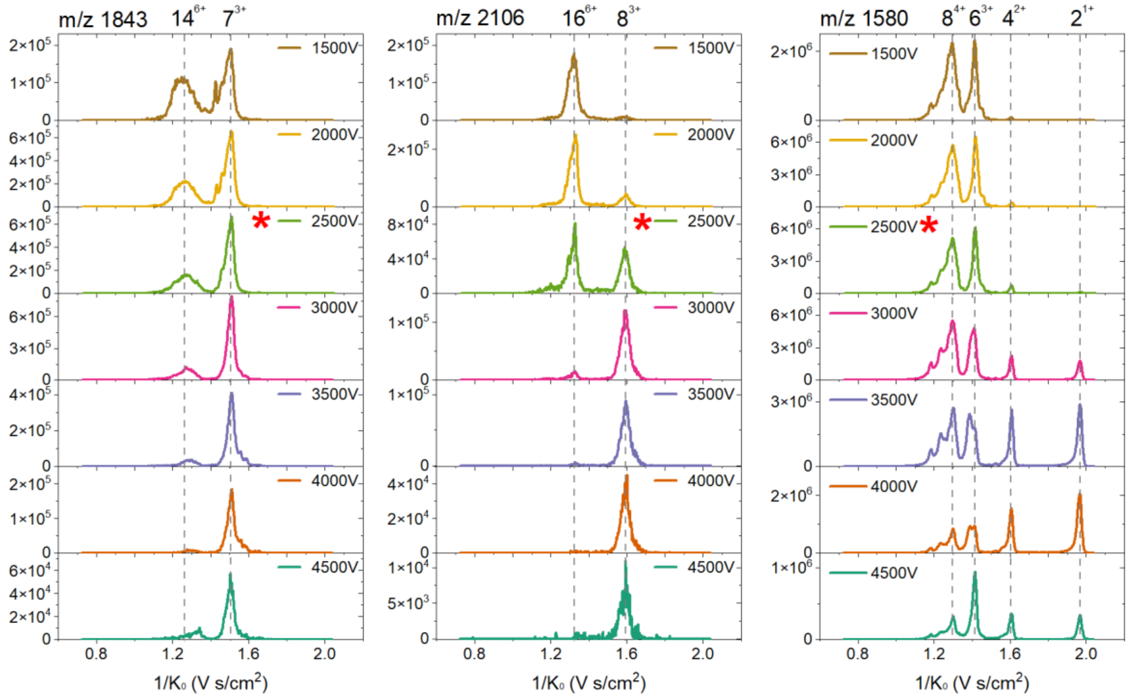

Figure S1. Extracted ion mobility spectra of *m/z* 1843 (left), *m/z* 2106 (middle) and *m/z* 1580 (right). The peak assignments of the oligomers are indicated on top. As can be seen, the abundance of higher order oligomers decreases at high capillary voltage (≥3000 V). The red asterisk marks the capillary voltage that was used in the current study (2500 V).

Figure S2. Effect of D2 potential in the TIMS-Qq-ToF on the oligomer abundances

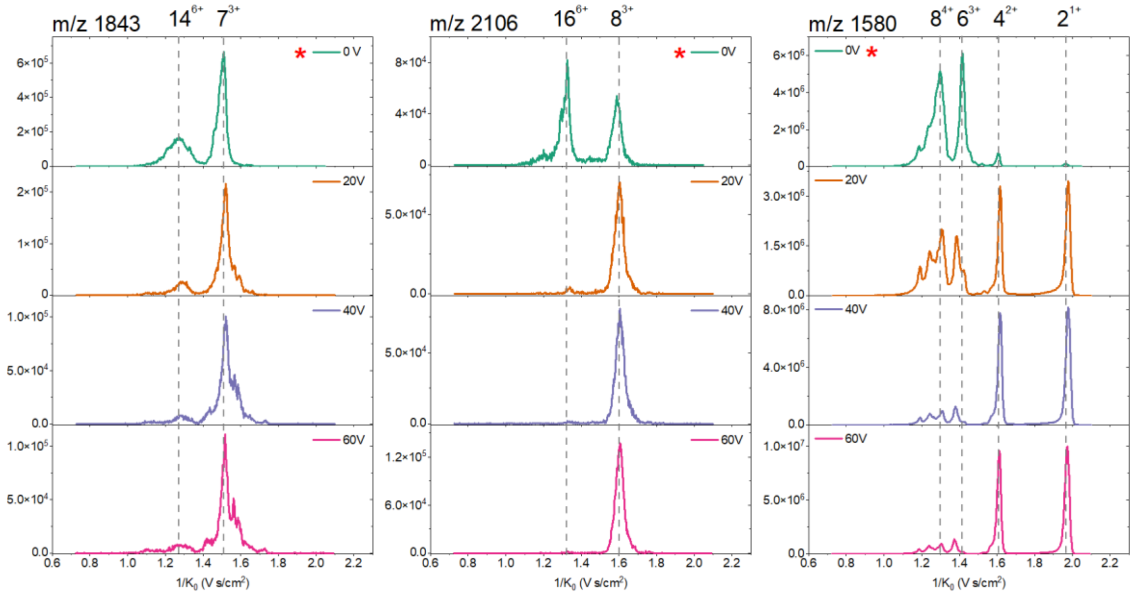

Figure S2. Extracted ion mobility spectra of *m/z* 1843 (left), *m/z* 2106 (middle) and *m/z* 1580 (right). The peak assignments of the oligomers are indicated on top. As can be seen, the abundance of higher order oligomers decreases when a high voltage is used (≥20V). The red asterisk marks the D2 voltage that was used in the current study (0 V).

Figure S3. Effect of D3 potential in the TIMS-Qq-ToF on oligomer abundances

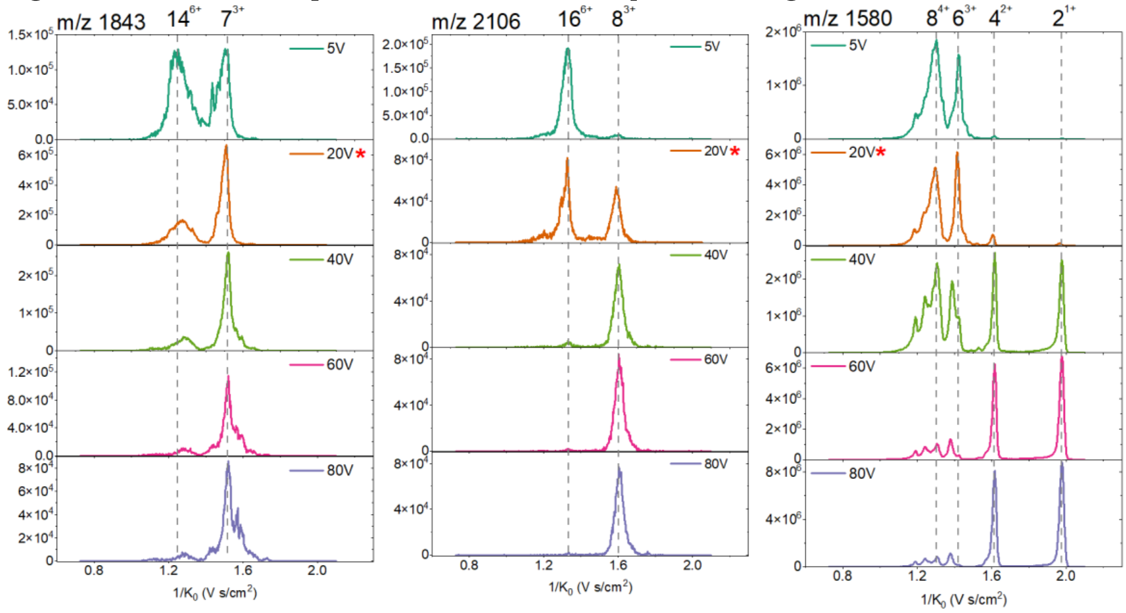

Figure S3. Extracted ion mobility spectra of *m/z* 1843 (left), *m/z* 2106 (middle) and *m/z* 1580 (right). The peak assignments of the oligomers are indicated on top. As can be seen, the abundance of higher order oligomers decreases when a high voltage is used (≥40V). However, when the voltage is too low (5V), the abundance of the lower charged oligomer is significantly reduced as can be observed for 8^{3+} (*m/z* 2106) and 2^{1+} (*m/z* 1580)The red asterisk marks the D3 voltage that was used in the current study (20V).

Figure S4. Effect of D6 potential in the TIMS-Qq-ToF on oligomer abundances

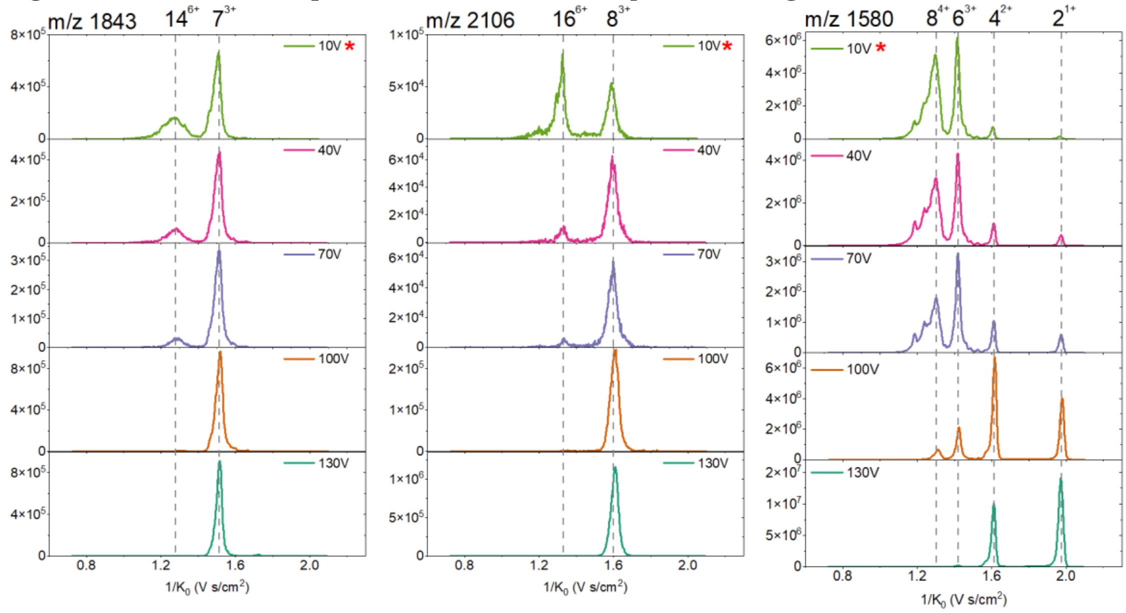

Figure S4. Extracted ion mobility spectra of *m/z* 1843 (left), *m/z* 2106 (middle) and *m/z* 1580 (right). The peak assignments of the oligomers are indicated on top. As can be seen, the abundance of higher order oligomers decreases when a high voltage is used (≥70V). The red asterisk marks the D6 voltage that was used in the current study (10 V).

Figure S5. Effect of ion energy and collision energy on fragmentation in the collision cell

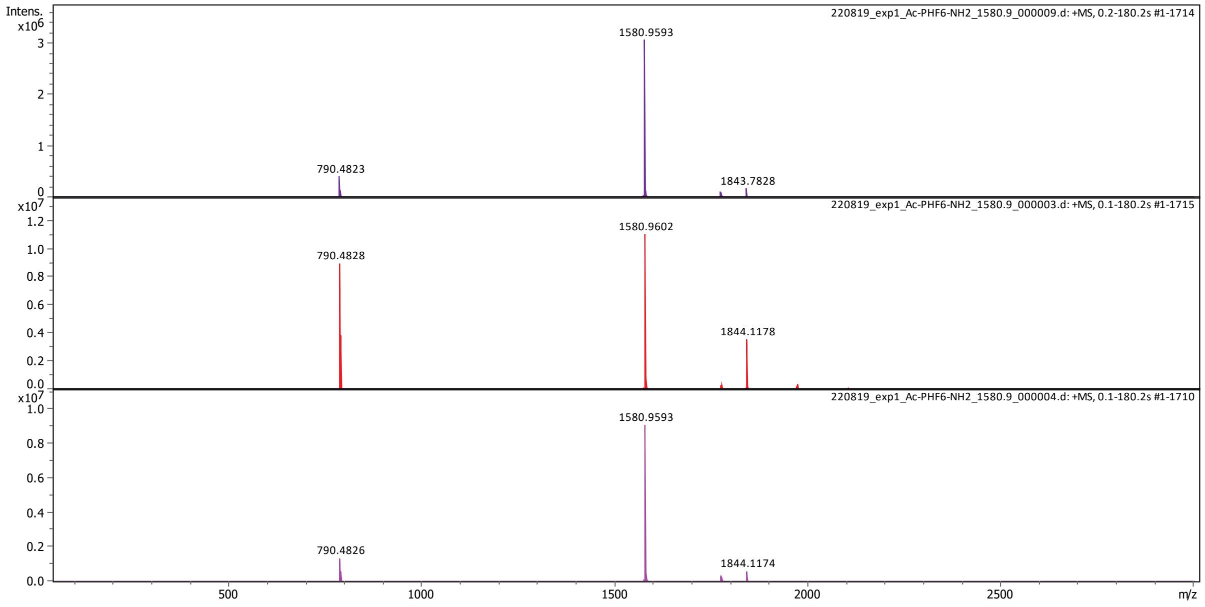

Figure S5. Mass spectra of the quadrupole-filtered *m/z* 1580 measured at different ion/collision energies: top (6/3 eV), middle (15/0 eV) and bottom (9/0 eV). The harsher settings (15/0 eV, red) produce more fragments. The settings at the bottom (9/0 eV) resulted in more transmission and did not cause more fragmentation than the settings at the top (6/3 eV). Therefore, the 9/0 eV ion/collision energy settings were used for the current study.

Table S2. Instrumental parameters on The Photo-Synapt for TWIMS IM-MS measurements

Two methods are shown, where the normal method represents the settings for Figure 5A in the main text with harsher source voltages and different ion mobility parameters and the softer settings were used in the comparison with TIMS and CCS elucidation (also main text, Figure 5B). Parameters highlighted in red are those that were different for the two set of settings for the TWIMS measurements.

	**Normal settings**	**Soft settings**
	Source	
Mass Lynx Tune Page name		
Capillary voltage	*2.1 kV*	*1.8 kV*
Sampling cone	*30 V*	*10 V*
Extraction cone	*6 V*	*3 V*
Source temperature	*80 °C*	*80 °C*
Desolvation temperature	*150 °C*	*150 °C*
Source gas flow	*0 mL/min*	*0 mL/min*
Cone gas flow	*0 L/h*	*0 L/h*
Desolvation gas flow	*500 L/h*	*500 L/h*
	Instrument parameters	
Trap CE	*4 V*	*4 V*
Transfer CE	*0 V*	*0 V*
Trap gas flow	*0.8 mL/min*	*2 mL/min*
Helium Cell gas flow	*180 mL/min*	*180 mL/min*
IMS gas flow	*60 mL/min*	*50 mL/min*
	TriWave DCs	
	Trap DC	
Entrance	*3 V*	*3 V*

Bias	*45 V*	*35 V*
Trap DC	*-2 V*	*0 V*
Exit	*0 V*	*0 V*
IMS DC		
Entrance	*25 V*	*25 V*
Helium cell DC	*35 V*	*35 V*
Helium Exit	*-5 V*	*-5 V*
Bias	*3 V*	*3 V*
Exit	*0 V*	*0 V*
Tri Wave		
Source		
Wave velocity	*200 m/s*	*200 m/s*
Wave height	*0.2 V*	*0.2 V*
Trap		
Wave velocity	*313 m/s*	*313 m/s*
Wave height	*6 V*	*6 V*
IMS		
Wave velocity	*300 m/s*	*800 m/s*
Wave height	*30 V*	*30 V*
Transfer		
Wave velocity	*190 m/s*	*190 m/s*
Wave height	*4 V*	*4 V*
Vacuum		
IMS	*2.73 mbar*	*2.92 mbar*
RF		
Source RF Offset	*350 Vpp*	*350 Vpp*
IMS RF Offset	*350 Vpp*	*350 Vpp*
IMS Mobility RF Offset	*250 Vpp*	*250 Vpp*
Trap RF Offset	*300 Vpp*	*300 Vpp*
Transfer RF Offset	*380 Vpp*	*380 Vpp*
Ion Maiden Settings		
Lens1	*185 V*	*180 V*
Pins1	*185 V*	*184 V*
Trap1	*185 V*	*184 V*
Lens2	*190 V*	*184 V*
Pins2	*185 V*	*184 V*
Trap2	*300 V*	*184 V*
Lens3	*260 V*	*260 V*
IMS	*170 V*	*170 V*
Hex1	*179.25 V*	*184 V*
Hex2	*179.75 V*	*184 V*
RF hex1	*800 Vpp*	*700 Vpp*

Section S1. CCS Calibration Procedure for Photo-Synapt (TWIMS) Data

Since TWIMS instruments have a non-static electric field, direct measurement of collision cross section (CCS) values is not straightforward and requires for the calibration procedure to have calibrants with known CCS values measured under the same experimental settings as the analyte molecules whose CCS values need to be determined. Numerous CCS calibration protocols were reported in the literature before[1–6]. Here, we have used the power law relationship between the CCS values and drift times. It should be noted that the power law relationship was derived by measuring a large number of different molecules with known CCS values, which turned out to be proportional to t_D^X, where t_D is the drift time and X is an empirically determined parameter[1]. However, there are now more advanced calibration protocols that take into account ion motions and can significantly reduce calibration errors[7].

The choice of calibrants is very important in the calibration protocol. It is recommended to use calibrants that are of the same molecular class and cover the same charge states and CCS (mobility) values as the analytes molecules[8,9]. Polyalanine is commonly used to create a calibration curve for peptides[10]. We used Agilent ESI tuning mix[11] and denatured ubiquitin[2] and their CCS values measured on a drift tube with N_2 to make the calibration curve and determine the CCS values of the analytes. Although the tuning mix is singly charged, it covers a wide range of *m/z* and drift time distributions. This calibrant was also used in the TIMS experiments. Ubiquitin, on the other hand, covers higher charge states of the analytes.

First, the drift times must be corrected for the *m/z* dependent time that ions spend after leaving the ion mobility cell and before reaching the detector. This requires the determination of an empirical constant called the enhanced duty cycle (EDC) delay coefficient in the MassLynx. For the modified Photo-Synapt, this was estimated by measuring the drift time of tuning mix and denatured ubiquitin, cytochrome C and myoglobin. Their CCS values were determined from a calibration curve and the EDC that gave the minimum error was chosen as optimal. In our case, the optimal EDC for the experimental settings reported here was found to be 90. The minimum error was approximately 1 %. The corrected drift times t'_D were determined as follows:

$$t'_D = t_D - 0.001 * EDC * \sqrt{m/z} \tag{1}$$

where t_D is the measured drift time.

The CCS values of the calibrants measured on the drift tube (CCS^{DT}) were corrected for the charge state *z* and the reduced mass μ.

$$CCS' = \frac{CCS^{DT}}{z\,(1/\mu)^{1/2}} \tag{2}$$

where $1/\mu = 1/m_I + 1/m_N$ with m_I is the mass of the ion and m_N is the mass of the neutral gas.

The plot of $ln(CCS')$ against $ln(t'_D)$ was fitted with a linear relationship of the following form:

$$ln(CCS') = X * ln(t'_D) + ln(A) \tag{3}$$

where *X* and *A* are the slope and the intercept of the linear fit that were used to calculate the CCS values of the analytes. The R^2 values of the linear fit were greater than 0.99 for the calibrants used in the current study.

Then, the analytes were measured with the same experimental parameters as the calibrants, and the drift times of the analytes were corrected using equation *(1)*. Finally, the CCS values of the analytes CCS^{EST} were estimated using the following equation:

$$CCS^{EST} = A * z * (t'_D)^X * (1/\mu)^{1/2} \tag{4}$$

The calibration curves as well as the estimation of the CCS values were done in Excel spreadsheets that were downloaded from the website of the group of Prof. Dr. Kevin Pagel (Freie Universität Berlin). (https://www.bcp.fu-berlin.de/en/chemie/chemie/forschung/OrgChem/pagel/research/carbohydrates/index.html)

Figures S6-S7. ThT fluorescence assays for PHF6 peptides

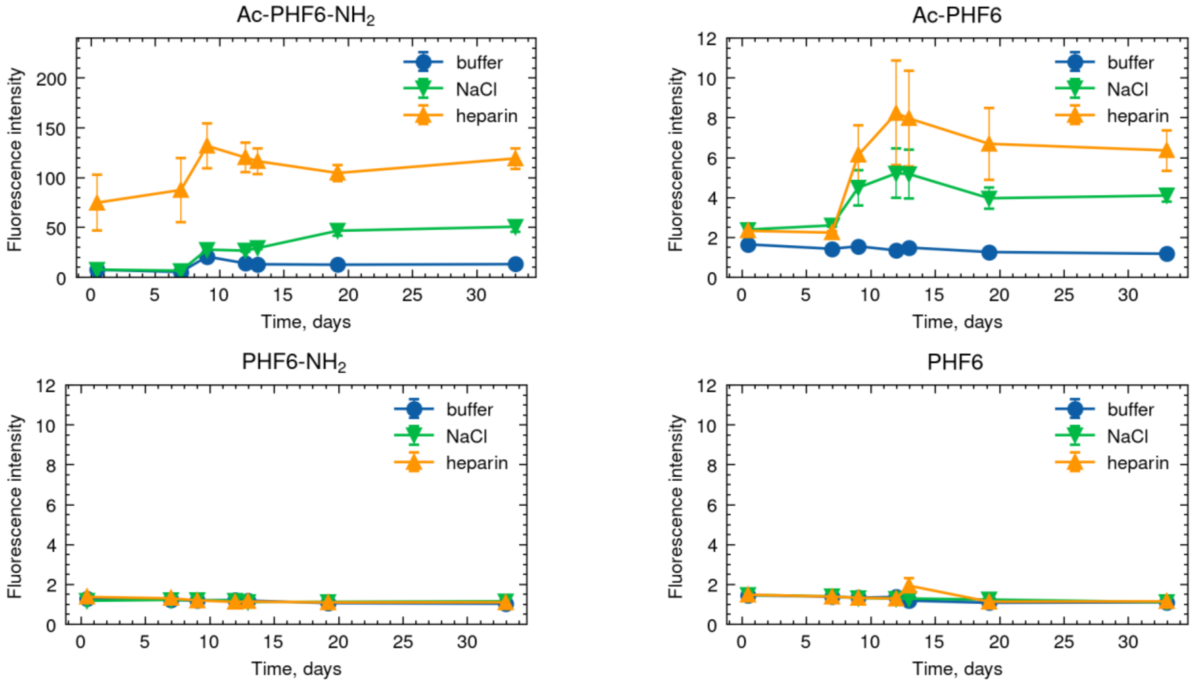

Figure S6. Average fluorescence intensity in arbitrary units as a function of time for 150 μM solutions incubated at 37 °C of the capped peptides Ac-PHF6-NH_2, Ac-PHF6, PHF6-NH_2, and PHF6 under three different conditions: in 10mM ammonium acetate (AA) buffer (blue), with addition of 150mM NaCl (green) or 1.15μM heparin (orange). The data for the first time were averaged once over the whole measurement of 22 hours. Error bars indicate the standard deviation divided by 3 because 9 independent experiments were conducted for every late time point. The y-axis for Ac-PHF6-NH_2 is 20 times larger than for the rest of the peptides.

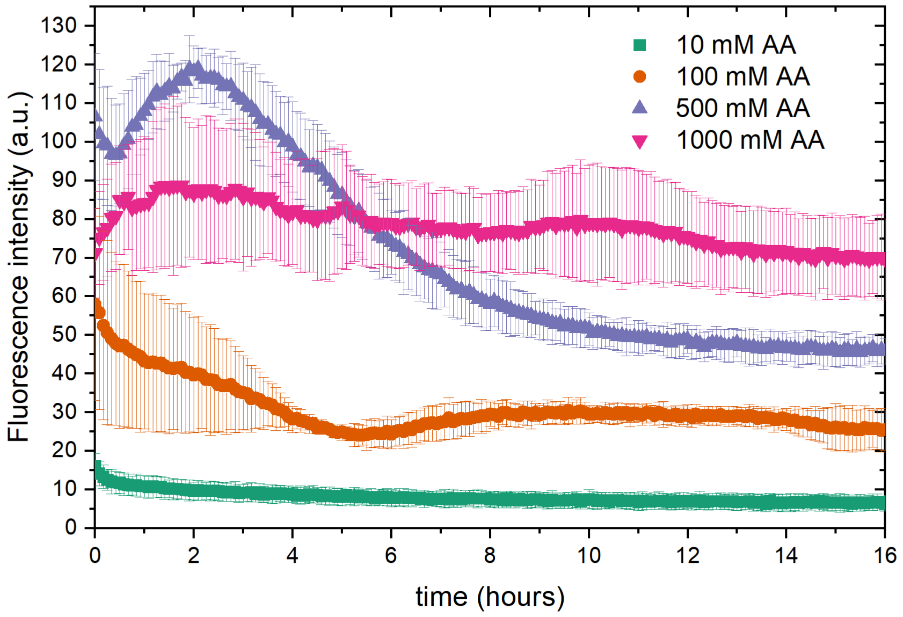

Figure S7. Average fluorescence intensity in in arbitrary units as a function of time for the Ac-PHF6-NH_2 peptide (150μM) in four different concentrations of ammonium acetate (AA) buffer solutions 10, 100, 500 and 1000mM (all solutions had pH ~7.4), shown in green, orange, pink, and purple, respectively. Data were averaged over 3 wells. Error bars indicate the standard deviation. Here, the measurements were done with the same plate reader settings as described in the main text. The well plate was measured at 37 °C for 16 hours every 5 minutes without shaking between readings.

Figure S8. TEM images of Ac-PHF6-NH_2 peptide

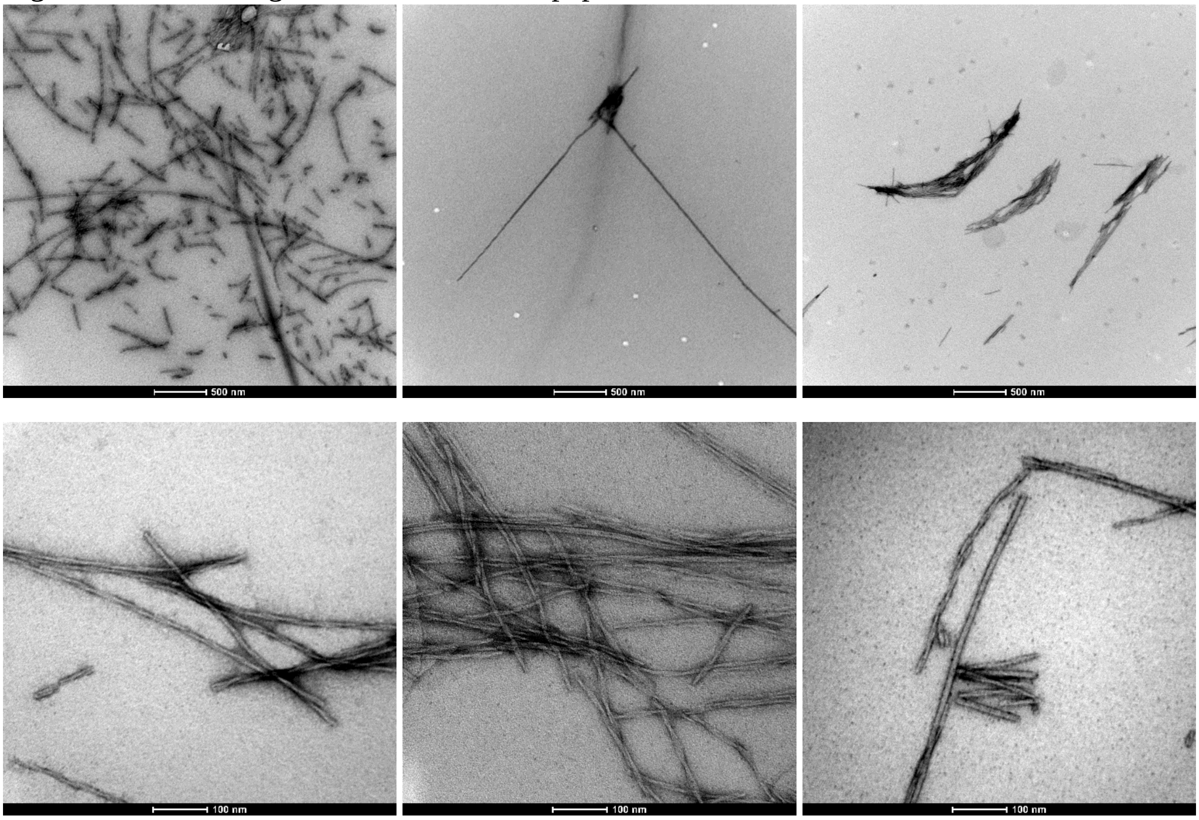

Figure S8. TEM images of the fibrillar structures obtained after 9 days incubation at 37 °C of the 150 µM Ac-PHF6-NH_2 in 10 mM ammonium acetate buffer (left) or buffer enriched with either 150 mM NaCl (middle) or 1.15 µM heparin (right). The top row of images gives an overview of the aggregates in the sample and the bottom row shows the aggregates at a higher magnification. The scale bar in the top row is 500nm and in the bottom row 100nm.

For the Ac-PHF6-NH_2 peptide incubated with only 10 mM AA buffer, fibrillar structures of different lengths (100nm to 2 µm) can be observed. The shorter fibrils can be explained by the fragmentation, i.e. the fibrils were broken into smaller fragments during sample preparation. The higher magnification image shows that the fibrils are twisted.

The Ac-PHF6-NH_2 sample incubated with 150 mM sodium chloride shows a few long fibrils (more than 2 µm) in the overview image. The higher magnification image indicates that most of the fibrils again have a twisted morphology and are longer than in the first condition with only buffer.

For the last condition, where Ac-PHF6-NH_2 was incubated with 1.15 µM heparin, groups (tangles) of fibrils and a few shorter isolated fibrils were imaged. The fibrils in the tangles vary in size and their morphology is difficult to determine from the overview image. The enlarged image (bottom) shows straight fibrils and fibrils with two twisted strands. The straight and twisted fibrils have a similar diameter (about 20 nm).

When comparing the structures observed for the three conditions, differences can be seen. For the sample incubated in buffer, more and shorter fibrils were observed, whereas incubation with sodium chloride resulted in less abundant but longer fibrils. This can be explained by the stabilizing effect of salts on fibrils[12]. For heparin, the fibrils were grouped closer together than for the other two samples.

The last two conditions for 150 µM Ac-PHF6-NH_2 with 150 mM NaCl and 1.15 µM heparin were previously measured with TEM after 14 days incubation at 37 °C by Arya et al.[13]. They also observed fibrils for both conditions, but without heparin Ac-PHF6-NH_2 formed mostly straight filaments, while

in our experiment we observed mostly twisted fibrils with NaCl. With heparin, we observed twisted filaments, which was also the case in the previous study.

Figure S9. TEM images of PHF6 peptides

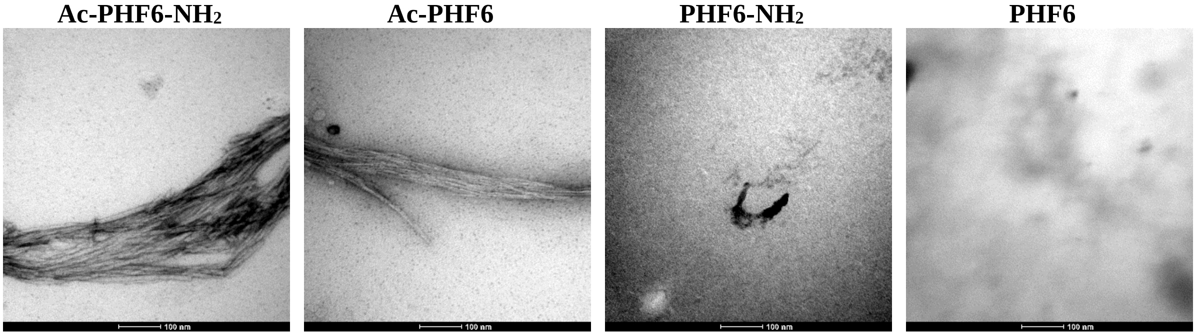

Figure S9. TEM images of the fibrillar structures obtained after 9 days incubation at 37 °C of the 150 μM peptide solutions in 10 mM ammonium acetate with 1.15 μM heparin of Ac-PHF6-NH_2, Ac-PHF6, PHF6-NH_2, and PHF6. The scale bar is 100nm.

For both PHF6 and PHF6-NH_2 no aggregates were observed. The samples with Ac-PHF6 and Ac-PHF6-NH_2 show fibrillar structures. These are tightly clustered, making it difficult to measure their length and diameter, and determine differences in morphology. In general, the absence or presence of fibrillar structures for the four different PHF6 capping variants is consistent with the fluorescence assay shown in Figure S6. There, PHF6 and PHF6-NH_2 samples show no increase in fluorescence, thus no formation of fibrils could be detected, whereas fibril formation could be observed for Ac-PHF6 and Ac-PHF6-NH_2. In the work of Arya et al.[13] the fibrils were observed not only for Ac-PHF6 and Ac-PHF6-NH_2, but also for PHF6-NH_2. This can be explained by their longer incubation time (14 days), which gives the sample more time to aggregate. They could also detect morphological differences between the samples. In the images described here, no morphological differences could be detected because the resolution of the images was lower.

Figure S10. Ion mobility assignment of fragment ions due to neutral loss of monomer

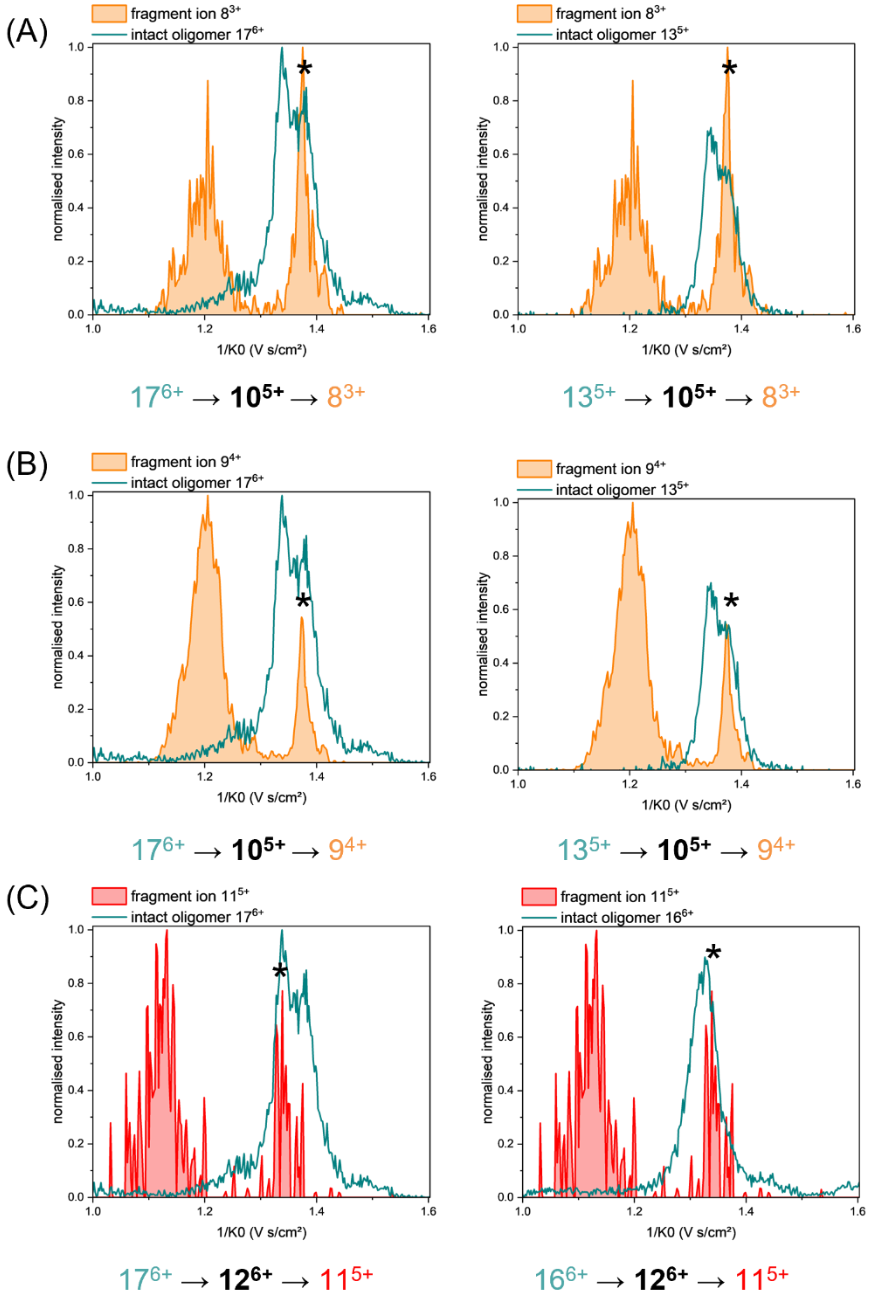

Figure S10. Ion mobility spectra of fragment ions resulting from the fragmentation of *m/z* 1580 in the collision cell: (A) the fragment ion 8^{3+} (shown in yellow) and the intact oligomers 17^{6+} and 13^{5+} (shown in green), (B) the fragment ion 9^{4+} (shown in yellow) and the intact oligomers 17^{6+} and 13^{5+} (shown in green), (C) the fragment ion 11^{5+} (shown in yellow) and the intact oligomers 17^{6+} and 16^{6+} (shown in green). Possible fragmentation pathways are shown at the bottom of the figures.

The intact oligomers (shown in green) can first dissociate at the TIMS-multipole interface into ions with *m/z* 1580, i.e. $10^{5+}$and 12^{6+} (shown in bold in the fragmentation pathway), mainly due to neutral monomer loss These ions with *m/z* 1580 are then selected by the quadrupole, and then further fragmented in the collision cell into the fragment ions (8^{3+}, 9^{4+}, and 11^{5+}, shown in yellow and red). Thus, the features of the intact oligomers at about 1.33 and 1.37 V·s/cm^2 are preserved and appear as additional peaks (marked with asterisks) in the ion mobility spectra of the fragment ions formed in the collision cell.

Figure S11. Comparison of mass spectra with normal and soft settings on the Photo-Synapt

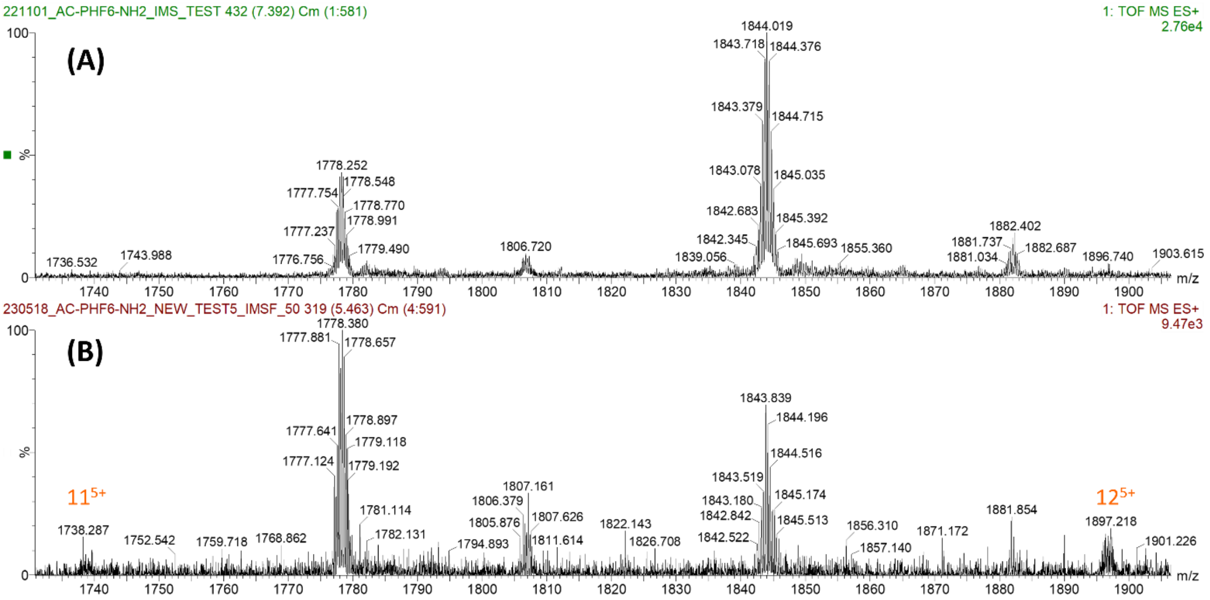

Figure S11. Averaged mass spectra of Ac-PHF6-NH_2 recorded over 10 minutes with normal (A) and softer (B) settings on the Photo-Synapt showing the region where oligomers are present. With softer settings the presence of two oligomers 11^{5+} and 12^{5+} (annotated in orange) is clearly better than with normal settings.

Table S3 and Figure S12. Comparison of CCS values of Ac-PHF6-NH_2 peptide oligomers obtained by TIMS and TWIMS ion mobility techniques

This section shows the calculated CCS values of the Ac-PHF6-NH_2 peptide oligomers from the experiments using two different types of IM-MS techniques, TIMS-Qq-ToF (TIMS) and the Photo-Synapt (TWIMS). The averaged CCS values are listed below in Table S3 with their standard deviations. The comparison is made by calculating the relative difference between the two techniques in percent. If two conformers were measured on TIMS (shown in blue), and not found on the TWIMS instruments, the one that correlated better with the one measured on TIMS was included in the comparison.

Table S3. Comparison of CCS values determined on the TIMS and TWIMS IM-MS instruments for Ac-PHF6-NH_2 peptide oligomers.

m/z, monoisotopic	N, number of monomers	Z, charge state	TIMS CCS, Å^2	StDev	TWIMS CCS, Å^2	StDev	TIMS-TWIMS Relative error, %
Singly charged oligomers							
790.48	1	1	275	0.4	281	0.0	-2.2
1579.95	2	1	395	0.4	399	0.7	-1.1
Doubly charged oligomers							
395.75	1	2	313	0.4	302	0.0	3.6
790.48	2	2	464	0.9	464	0.0	0.1
1185.22	3	2	573	1.0	578	0.0	-0.8
1579.96	4	2	648	0.6	656	0.0	-1.2
1974.69	5	2	717	0.6	708	0.0	1.3
Triply charged oligomers							
1053.64	4	3	723	0.5	714	8.5	1.1
1316.79	5	3	792	0.7	799	0.0	-0.9
1579.96	6	3	857	0.9	862	0.0	-0.6
1843.12	7	3	910	0.2	923	3.8	-1.3
2106.27	8	3	958	0.8	983	1.5	-2.5
Quadruply charged oligomers							
1382.59	7	4	961	1.2	1047	0.0	
1382.59	7	4	1000	0.5	1047	0.0	-4.6
1579.96	8	4	1049	0.8	1056	0.0	-0.7
1777.33	9	4	1078	0.9	1113	0.0	
1777.33	9	4	1108	0.9	1113	0.0	-0.4
1974.69	10	4	1146	3.2	1166	0.0	-1.8
2172.06	11	4	1194	2.3	1219	0.0	-2.1
2369.43	12	4	1247	1.9	1283	0.0	-2.9
5+ charged oligomers							
1737.85	11	5	1273	1.1	1287	0.0	-1.1
1895.75	12	5	1320	1.3	1342	0.0	-1.6
2053.64	13	5	1349	0.8	1395	0.0	
2053.64	13	5	1388	1.8	1395	0.0	-0.5
2211.53	14	5	1423	1.8	1453	6.2	-2.0
2369.43	15	5	1474	2.3	1509	5.9	-2.4
2527.33	16	5	1530	3.0	1572	0.0	-2.7

6+ charged oligomers							
2106.27	16	6	1601	2.2	1620	8.2	-1.2
2237.85	17	6	1618	3.1	1682	15.6	
2237.85	17	6	1668	0.9	1705	22.9	-2.2
2369.43	18	6	1706	0.0	-		
2501.01	19	6	1764	2.9	1821	7.0	-3.2
2632.59	20	6	1817	9.4	1840	20.9	-1.3
						Average error (absolute value)	1.7

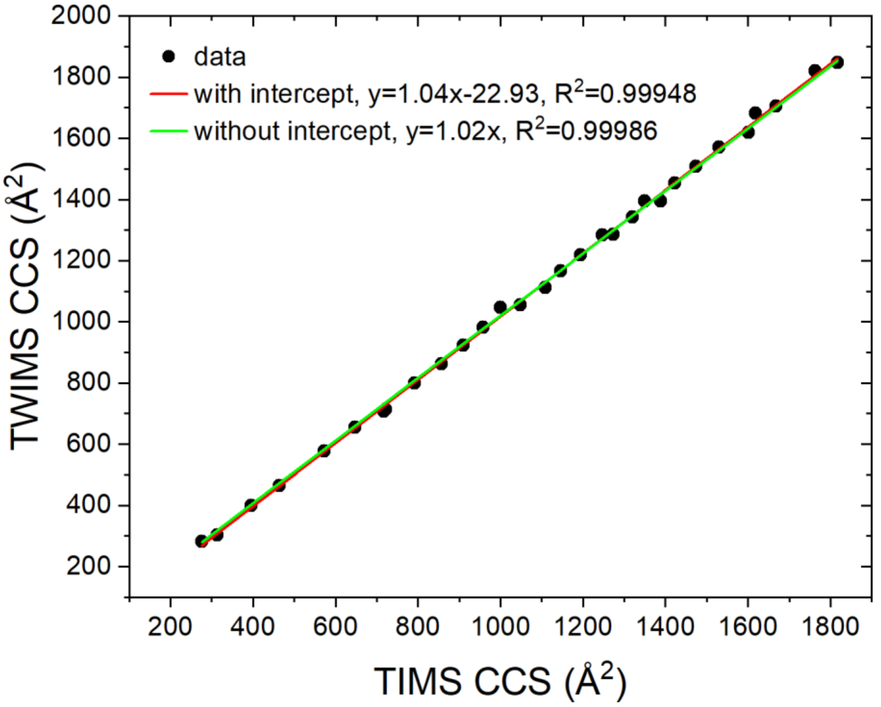

Figure S12. Correlation between the CCS values of Ac-PHF6-NH_2 peptide oligomers measured by TWIMS and TIMS IM-MS instruments. The green line shows the linear fit of the CCS values without intercept and the red line shows the linear fit with intercept.

Section S2. Ion Mobility Peak Assignments of the TIMS data

This section shows the details, the presented analysis, and the ion mobility peak assignments measured with the TIMS-Qq-ToF mass spectrometer. The ion mobilities of each *m/z* are shown with and without quadrupole selection of that mass peak. The extracted mass spectra from the ion mobility peaks show the charge states and allow to make the assignment. The *m/z* values that have multiple oligomers in the ion mobility are shown together in one section.

1. Doubly charged oligomers:

[1] *m/z* 395.8: 1^{2+}

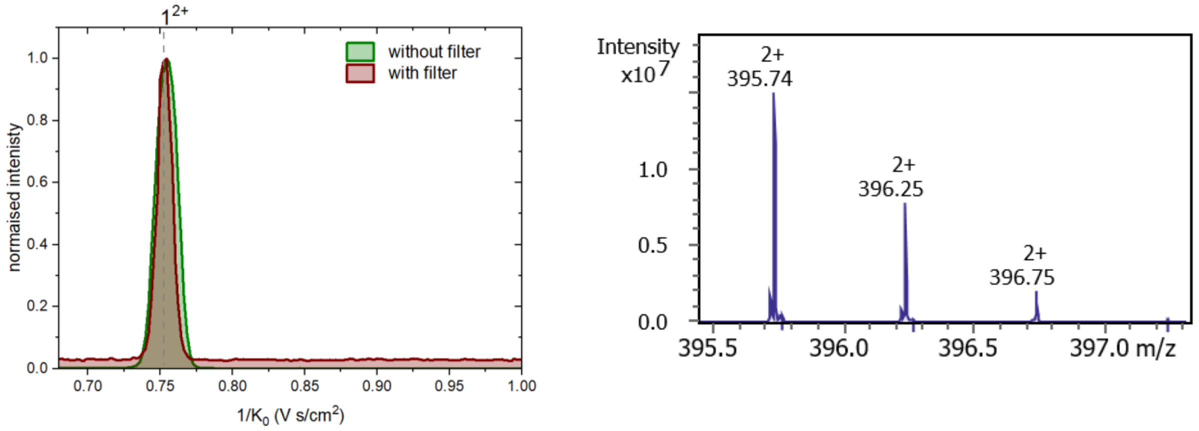

Figure D1. Left panel: Extracted ion mobility spectra of *m/z* 395.8 (1^{2+}) without quadrupole filter (green) and with quadrupole transmitting only selected *m/z* (red). The right panel shows the extracted mass spectrum from the mobility peak without quadrupole filter, showing the isotopic pattern of the doubly charged monomer.

[2] *m/z* 790.5: 1^{1+} and 2^{2+}

We assigned two peaks in the ion mobility spectrum for *m/z* 790.5. The peak at $1/K_0$=1.35 V·s/cm^2 corresponds to 1^{1+} and the small peak at $1/K_0$=1.15 V·s/cm^2 corresponds to 2^{2+}. The other peak at about 1.11 V·s/cm^2 in the quadruple filtered ion mobility is a fragment from a dimer with K+ adduct $[2M+K+H]^{2+}$.

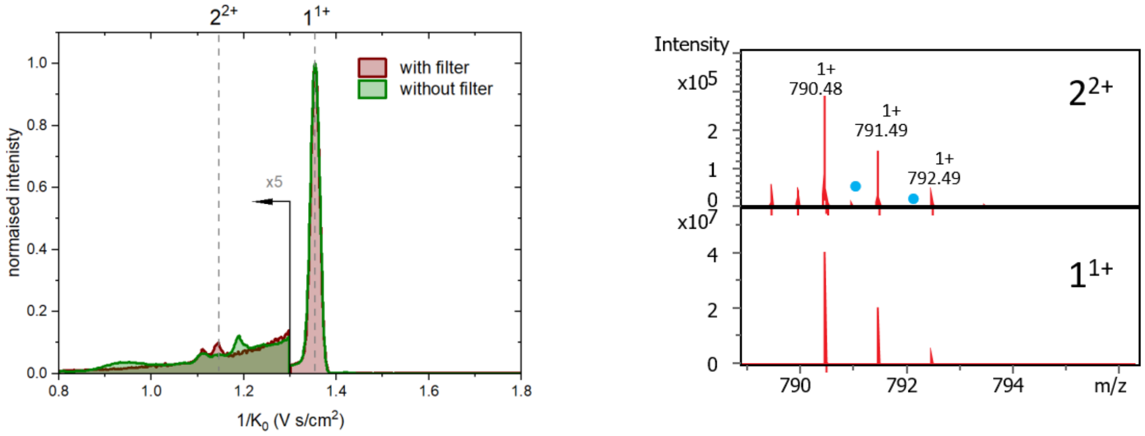

Figure D2. Extracted ion mobility spectra of *m/z* 790.5 (1^{1+} and 2^{2+}) without quadrupole filter (green) and with quadrupole transmitting only selected *m/z* (red). The right panel shows the extracted mass spectra of the mobility peaks without quadrupole filter. Blue circles indicate peaks corresponding exclusively to the doubly charged dimer.

[3] *m/z* 1185: 3^{2+} and 6^{4+}

The extracted ion mobility of *m/z* 1185, measured without quadrupole selection (green), shows a peak corresponding to the 4^{3+} oligomer (intact *m/z* 1054) in addition to the 3^{2+} and 6^{4+} oligomers, indicating that this 4^{3+} oligomer has fragmented into the 3^{2+} *m/z* channel. When *m/z* 1185 is quadrupole-selected, the 4^{3+} peak is still present in the quadrupole-selected, extracted ion mobility spectrum of *m/z* 1185 (red). This means that the 4^{3+} oligomer fragments into 3^{2+} after its mobility is measured (hence showing the $1/K_0$ value of the intact 4^{3+} oligomer) but before the quadrupole, since the 4^{3+} oligomer (*m/z* 1054) cannot pass through the quadrupole (set at the *m/z* 1185). We therefore assign two peaks in the ion mobility spectrum for *m/z* 1185. The peak at $1/K_0$=1.41 V·s/cm^2 corresponds to 3^{2+} and the peak at $1/K_0$=1.15 V·s/cm^2 corresponds to 6^{4+}. The peak at about $1/K_0$=1.19 V·s/cm^2 in the quadruple filtered ion mobility is a fragment from 4^{3+} which falls into 3^{2+} at the TIMS-multipole interface.

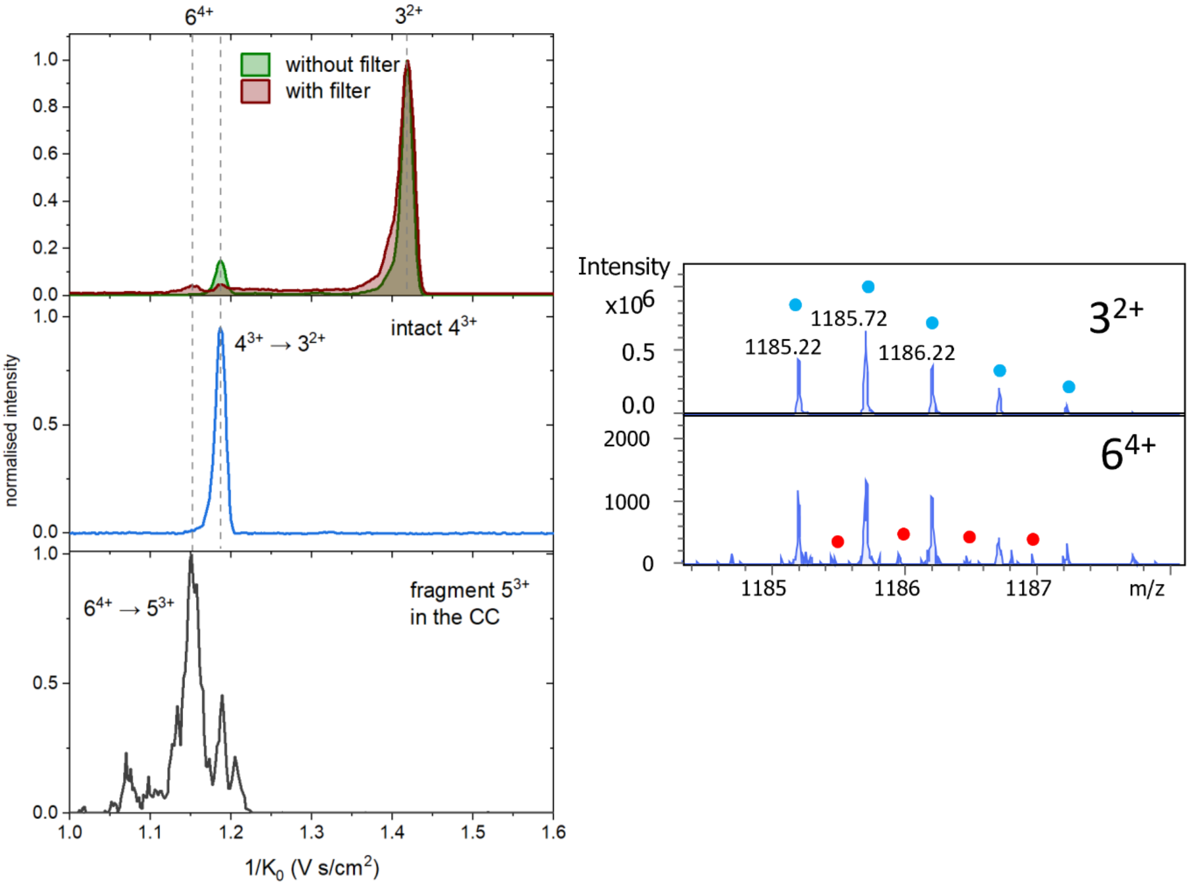

Figure D3. Extracted ion mobility spectra of *m/z* 1185 without quadrupole filter (green) and with quadrupole transmitting only selected *m/z* (red). The blue trace represents extracted ion mobility of intact 4^{3+} oligomer. The black trace at the bottom corresponds to the quadrupole selected extracted ion mobility spectrum of the 5^{3+} fragment resulting from the fragmentation of 6^{4+} in the collision cell (CC). The right panel shows the extracted mass spectra from the quadrupole filtered mobility peaks. Blue circles indicate peaks corresponding to the doubly charged oligomer 3^{2+}, while red circles highlight peaks corresponding exclusively to the oligomer 6^{4+}.

[4] *m/z* 1580: 2^{1+}, 4^{2+}, 6^{3+}, 8^{4+}, 10^{5+}, and 12^{6+}

We assign six peaks in the ion mobility spectrum for *m/z* 1580 based on the mobility of the fragment ions. The peak at $1/K_0$ = 1.959 V·s/cm^2 is singly-charged dimer 2^{1+}, $1/K_0$ = 1.606 V·s/cm^2 is 4^{2+}, $1/K_0$ = 1.416 V·s/cm^2– 6^{3+}, peak at $1/K_0$ = 1.299 V·s/cm^2 – 8^{4+}. The peaks at $1/K_0$ = 1.203 V·s/cm^2 corresponds to 10^{5+} and the peak $1/K_0$= 1.126 V·s/cm^2 is assigned as the 12^{6+} oligomer. The two features at around 1.15-1.25 V·s/cm^2 in the extracted ion mobility of *m/z* 1580 without quadrupole selection are originating from fragmentation into this mass channel in the collision cell.

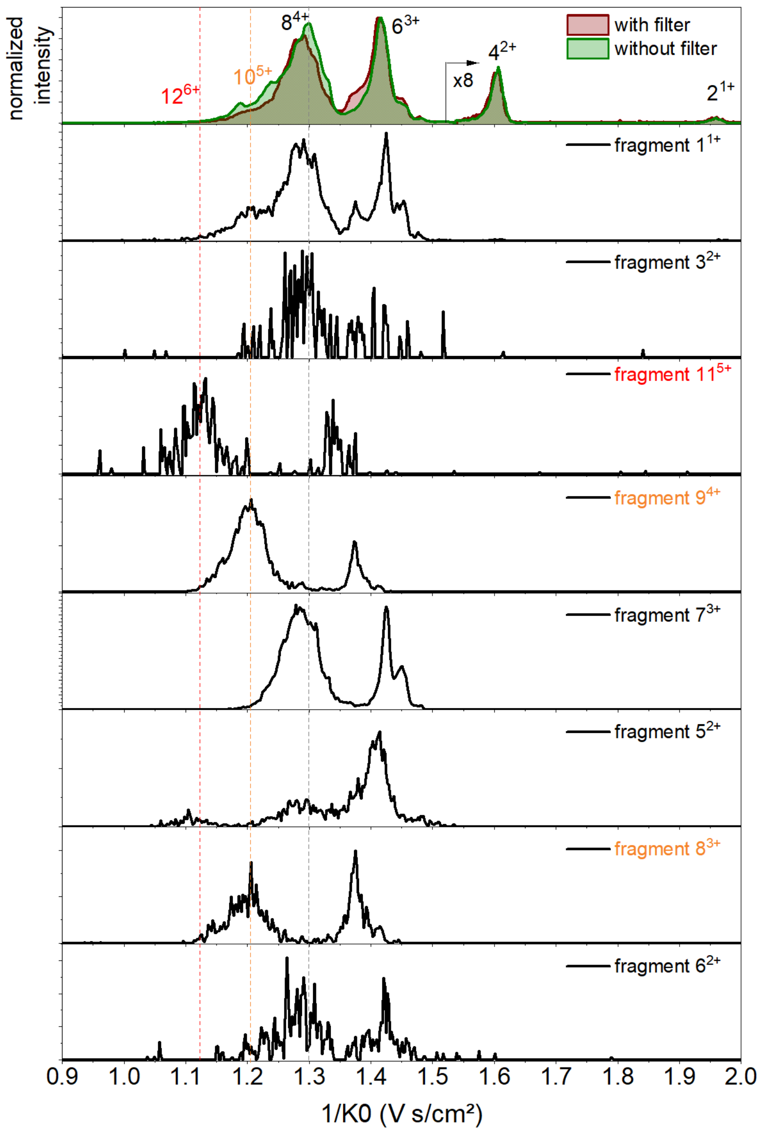

Figure D4. Extracted ion mobility spectrum of *m/z* 1580 without quadrupole filter (green) and total ion mobility spectrum with quadrupole transmitting only selected *m/z* (red). The region above 1.54 V·s/cm^2 was multiplied by 8 for clarity. The black traces correspond to the quadrupole selected extracted ion mobility spectra of fragments formed in the collision cell.

[5] *m/z* 1975: 5^{2+}, 10^{4+} and 15^{6+}

We assign three peaks in the ion mobility spectrum for *m/z* 1975. The peak at about $1/K_0$=1.8 V·s/cm^2 corresponds to 5^{2+}, the peak at $1/K_0$=1.42 V·s/cm^2 corresponds to 10^{4+}, and the peak at about $1/K_0$=1.28 V·s/cm^2 corresponds to 15^{6+}. The peak of 15^{6+} without quadrupole filter is shifted compared to the one with filter because of fragmentation occurring in the collision cell.

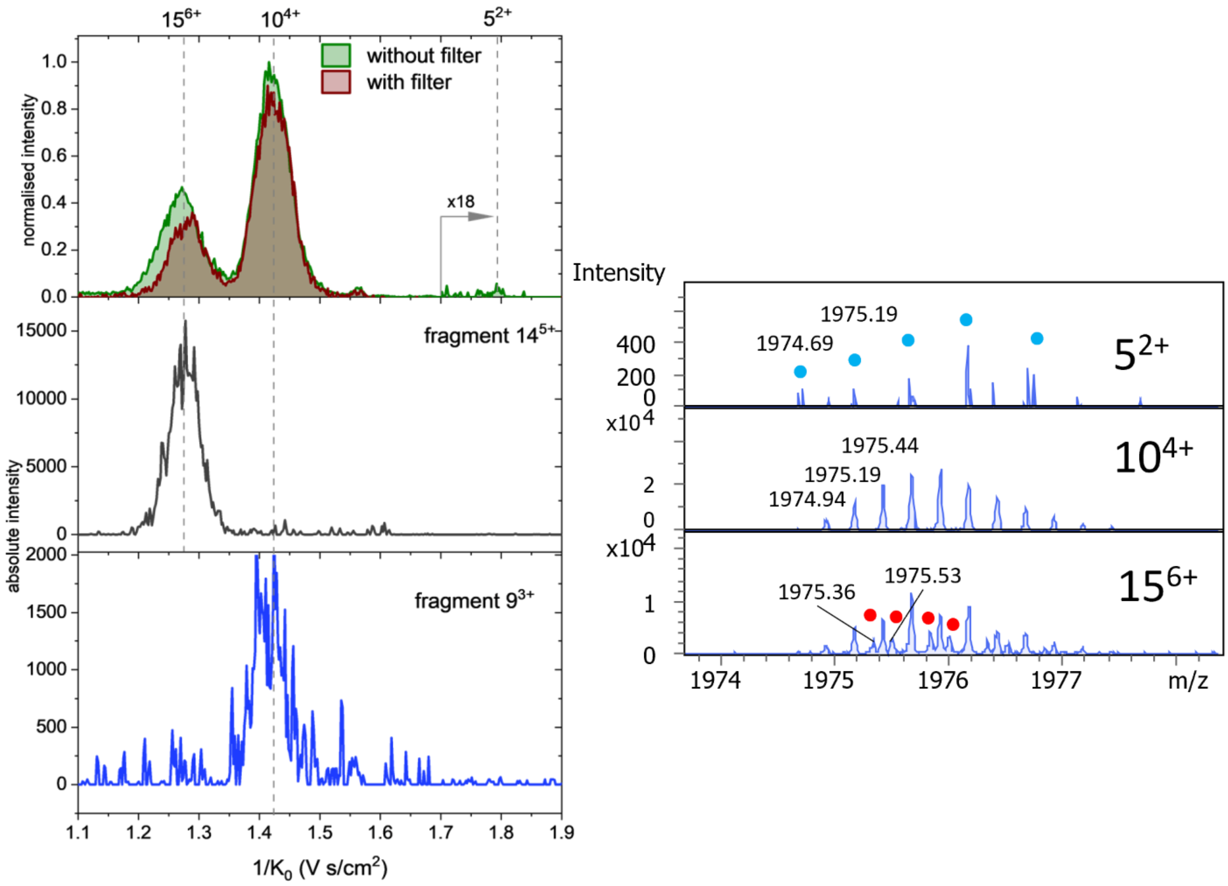

Figure D5. Extracted ion mobility spectra of *m/z* 1975 without quadrupole filter (green) and with quadrupole transmitting only selected *m/z* (red). The black trace corresponds to the quadrupole selected extracted ion mobility of 14^{5+} fragment resulting from the fragmentation of 15^{6+} in the collision cell. The blue trace corresponds to the quadrupole selected extracted ion mobility of 9^{3+} fragment resulting from the fragmentation of 10^{4+} in the collision cell. The right panel shows the extracted mass spectra from the mobility peaks without quadrupole filter. Blue circles indicate peaks corresponding to the doubly charged oligomer 5^{2+}, while red circles highlight peaks corresponding exclusively to the oligomer 15^{6+}.

2. Triply charged oligomers:

[1] *m/z* 1054: 4^{3+}

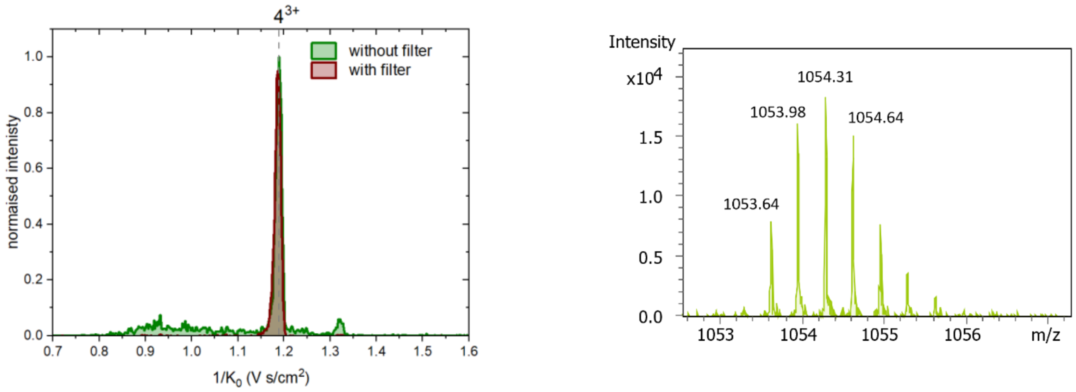

Figure T1. Extracted ion mobility spectra of *m/z* 1054 (4^{3+}) without quadrupole filter (green) and with quadrupole transmitting only selected *m/z* (red). The right panel shows the extracted mass spectrum from the mobility peak without quadrupole filter.

[2] *m/z* 1317: 5^{3+}

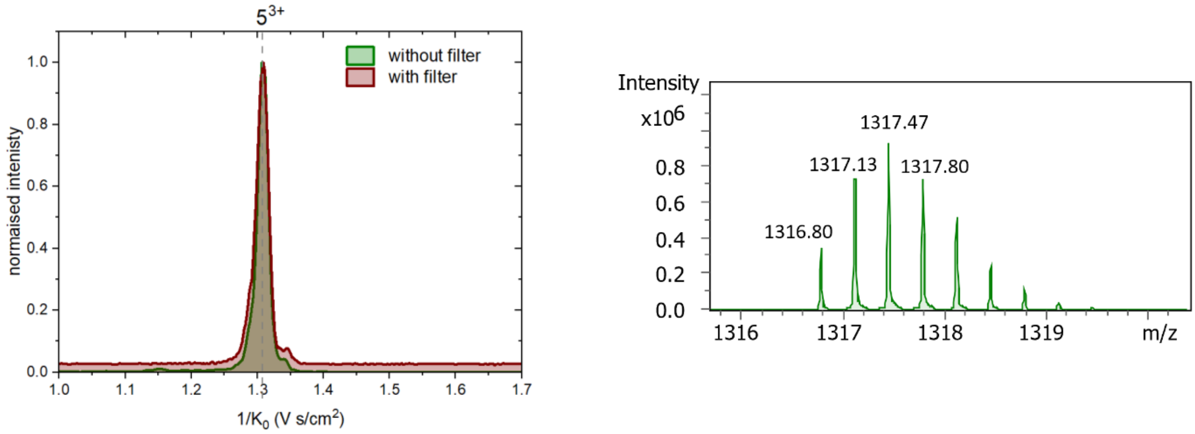

Figure T2. Extracted ion mobility spectra of *m/z* 1317 (5^{3+}) without quadrupole filter (green) and with quadrupole transmitting only selected *m/z* (red). The right panel shows the extracted mass spectrum from the mobility peak without quadrupole filter, showing a 3+ charge based on the isotopic distribution.

[3] *m/z* 1843: 7^{3+} and 14^{6+}

We assign two peaks in the ion mobility spectrum for *m/z* 1843. The peak at $1/K_0$=1.51 V·s/cm^2 corresponds to 7^{3+} and the peak at $1/K_0$=1.22 V·s/cm^2 corresponds to 14^{6+}. The peak assigned to 14^{6+} oligomer in the quadrupole selected ion mobilogram (red) looks narrower and shifted to the left compared to non-filtered extracted mobility (green). That is due to the fragmentation of 8^{4+} into 7^{3+} in the collision cell.

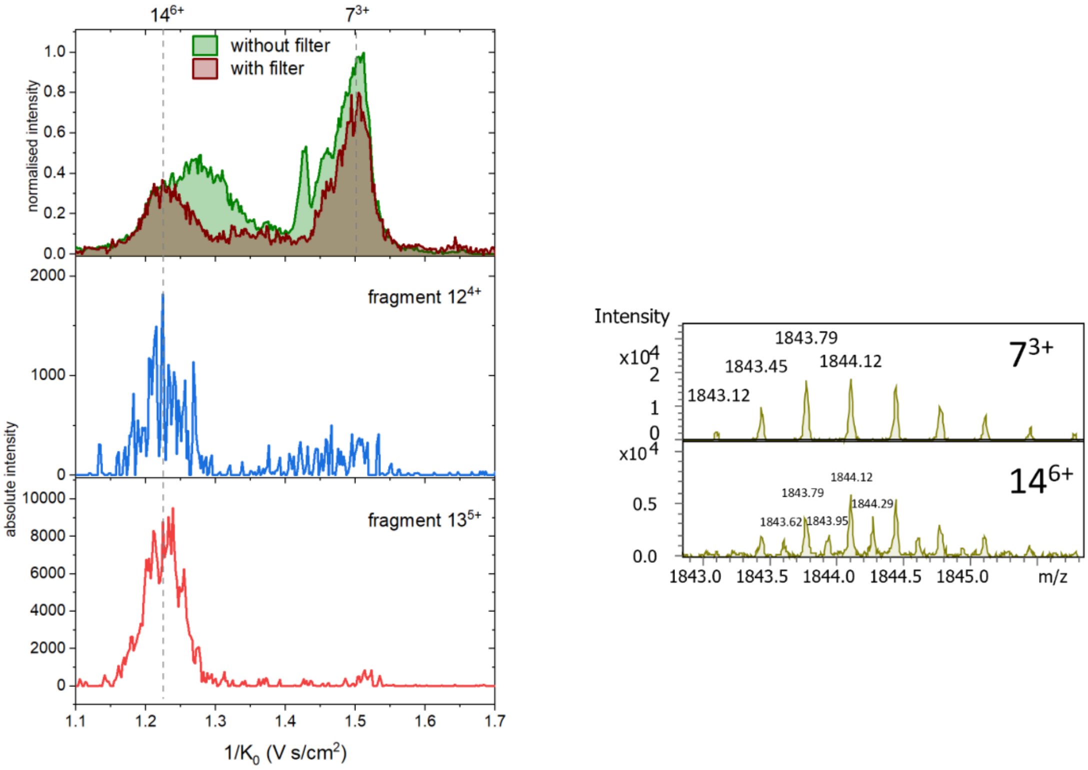

Figure T3. Extracted ion mobility spectra of *m/z* 1843 (7^{3+} and 14^{6+}) without quadrupole filter (green) and with quadrupole transmitting only selected *m/z* (red). The blue and red traces correspond to the quadrupole selected extracted ion mobility of the fragments resulting from the fragmentation of 14^{6+} in the collision cell. The right panel shows the extracted mass spectra from the mobility peaks without quadrupole filter.

[4] *m/z* 2106: 8^{3+} and 16^{6+}

We assign two peaks in the ion mobility spectrum for *m/z* 2106. The peak at $1/K_0$=1.59 V·s/cm^2 corresponds to 8^{3+} and the peak at $1/K_0$=1.32 V·s/cm^2 corresponds to 16^{6+}.

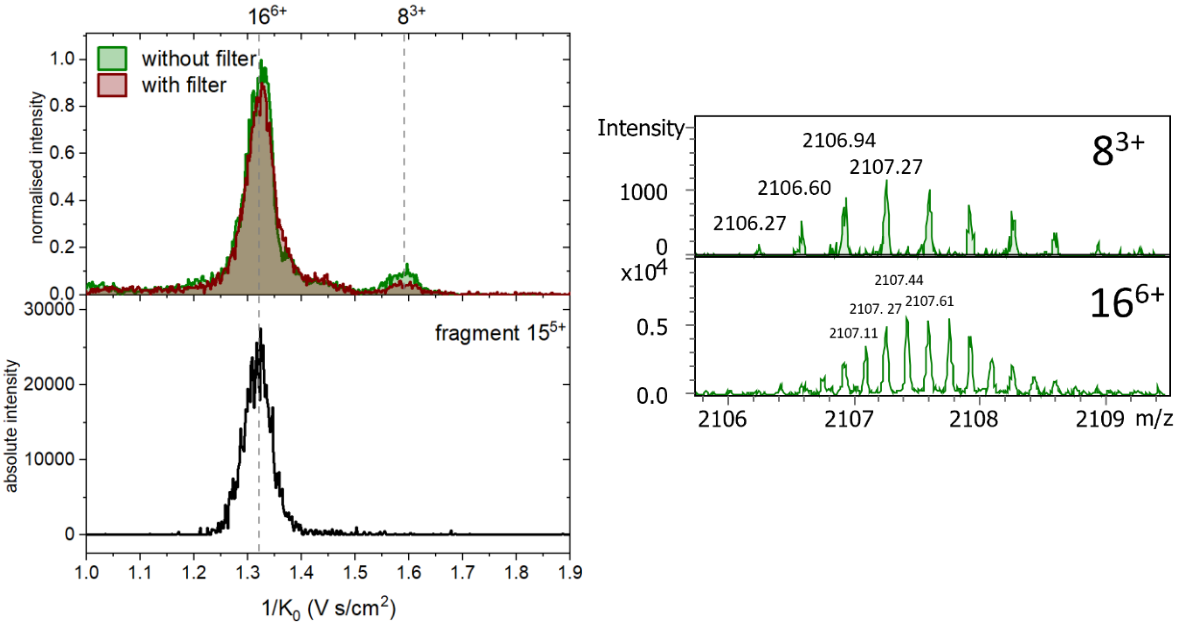

Figure T4. Extracted ion mobility spectra of *m/z* 2106 (8^{3+} and 16^{6+}) without quadrupole filter (green) and with quadrupole transmitting only selected *m/z* (red). The black trace corresponds to the quadrupole selected extracted ion mobility spectrum of the fragment 15^{5+} resulting from the fragmentation of 16^{6+} in the collision cell. The right panel shows the extracted mass spectra from the mobility peaks without quadrupole filter.

3. Quadruply charged oligomers:

[1] *m/z* 1383: 7^{4+}

We assign two peaks in the ion mobility spectrum of *m/z* 1383 at $1/K_0$=1.19 V·s/cm^2 and at $1/K_0$=1.24 V·s/cm^2 to two conformers of the 7^{4+} oligomer. Quadrupole section experiments did not show the presence of other higher order oligomers in this *m/z* channel. The peaks at about $1/K_0$=1.33 V·s/cm^2 are originating from fragments formed at the TIMS-multipole interface.

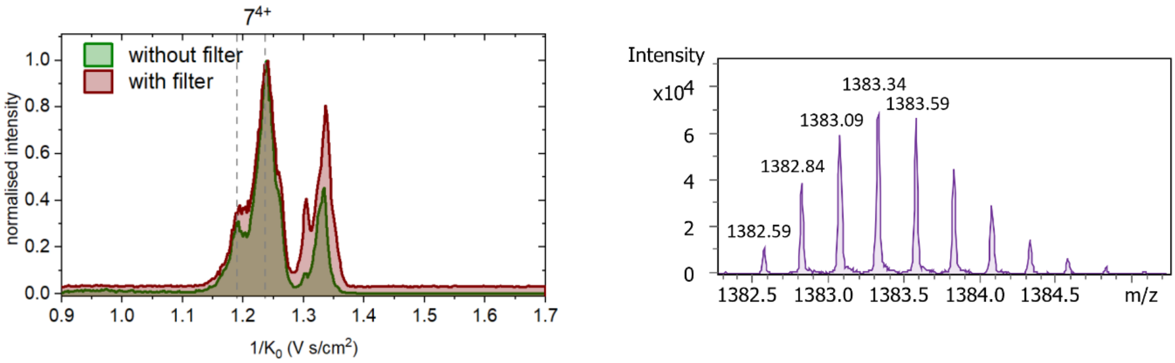

Figure Q1. Extracted ion mobility spectra of *m/z* 1383 (7^{4+}) without quadrupole filter (green) and with quadrupole transmitting only selected *m/z* (red). The right panel shows the extracted mass spectrum from the assigned mobility peaks without quadrupole filter.

[2] *m/z* 1777: 9^{4+}

We assign two peaks in the ion mobility spectrum of *m/z* 1777. The peaks at $1/K_0$=1.34 V·s/cm^2 and at $1/K_0$=1.38 V·s/cm^{2+} are assigned to the two conformations of the 9^{4+} oligomer since they are closely spaced. Quadrupole section experiments did not show the presence of other higher order oligomers in this *m/z* channel. The peak at around $1/K_0$=1.2 V·s/cm^{2+} in the non-filtered extracted mobility (green) correspond to the fragment of 10^{5+} into 9^{4+} that occurs in the collision cell.

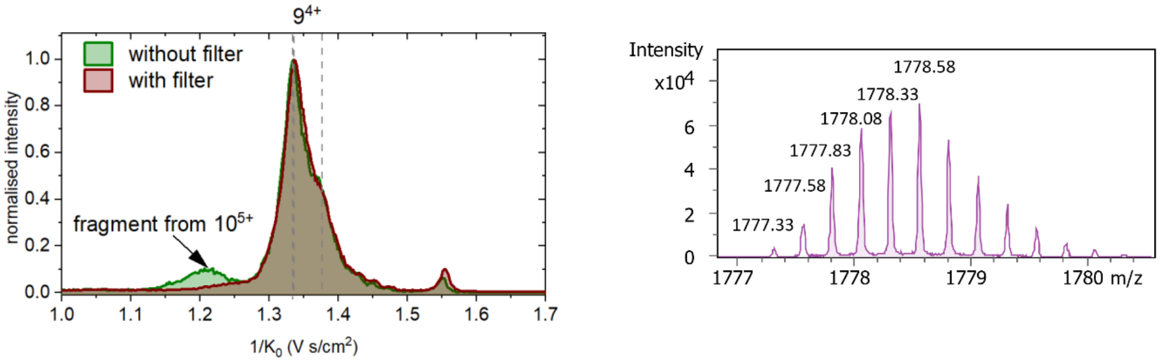

Figure Q2. Extracted ion mobility spectra of *m/z* 1777 (9^{4+}) without quadrupole filter (green) and with quadrupole transmitting only selected *m/z* (red). The right panel shows the extracted mass spectrum from the assigned mobility peaks without quadrupole filter.

[3] *m/z* 2172: 11^{4+}

We assign one peak in the ion mobility spectrum of *m/z* 2172 . The peak at $1/K_0$=1.48 V·s/cm^2 corresponds to the 11^{4+} oligomer. The broad peak in the non-filtered extracted mobility (green) at around $1/K_0$=1.3 V·s/cm^2 originates from fragmentation of 12^{5+} oligomer into 11^{4+} in the collision cell.

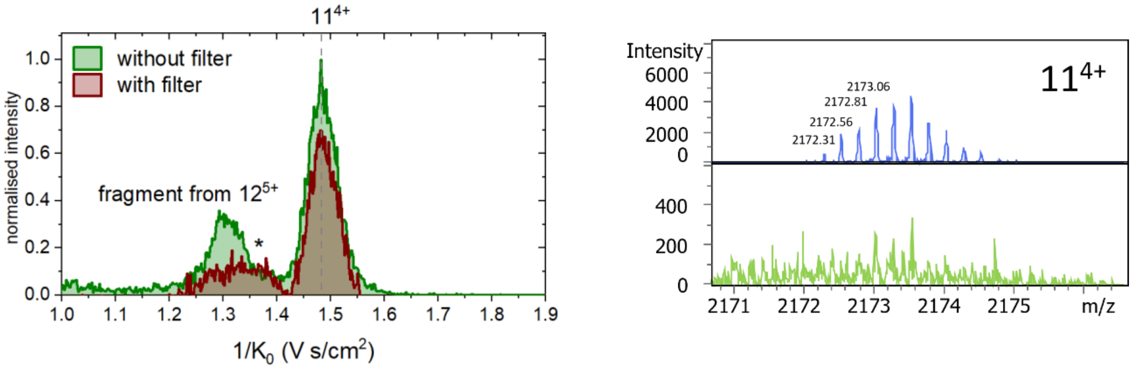

Figure Q3. Extracted ion mobility spectra of *m/z* 2172 (11^{4+}) without quadrupole filter (green) and with quadrupole transmitting only selected *m/z* (red). The right panel shows the extracted mass spectra from the mobility peaks with quadrupole filter. Top trace (blue) corresponds to the oligomer 11^{4+}, while the bottom one (green) could originate from the higher order oligomers that are present in the mobility peak marked with asterisk.

[4] *m/z* 2369: 12^{4+}, 15^{5+} and 18^{6+}

We assign three peaks in the ion mobility spectrum for *m/z* 2369. The peak at $1/K_0$=1.56 V·s/cm^2 corresponds to 12^{4+}, the peak at $1/K_0$=1.46 V·s/cm^2 corresponds to 15^{5+}, and the peak at $1/K_0$=1.41 V·s/cm^2 corresponds to 18^{6+}. Quadrupole section experiments didn't show the presence of other higher order oligomers in this *m/z* channel.

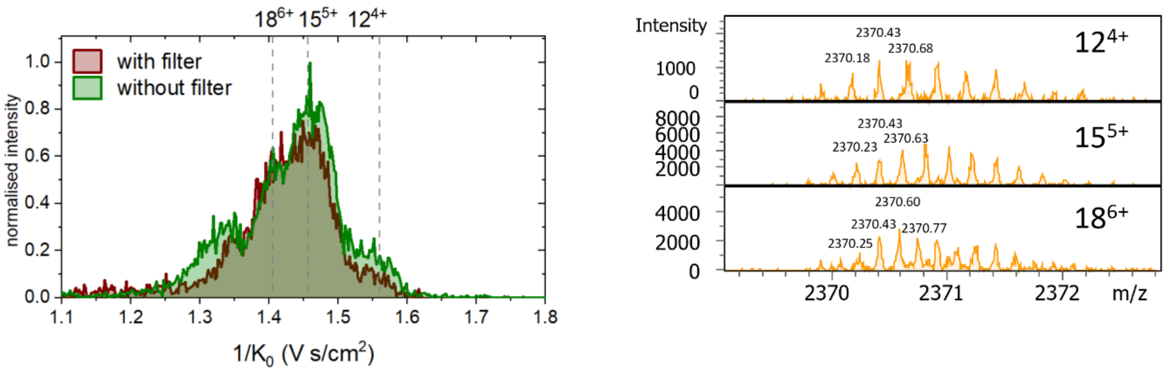

.

Figure Q4. Extracted ion mobility spectra of *m/z* 2369 (12^{4+}, 15^{5+}, and 18^{6+}) without quadrupole filter (green) and with quadrupole transmitting only selected *m/z* (red). The right panel shows the extracted mass spectra from the mobility peaks without quadrupole filter.

4. 5+ charged oligomers:

For all 5+ oligomers, quadrupole section experiments did not show the presence of other higher order oligomers.

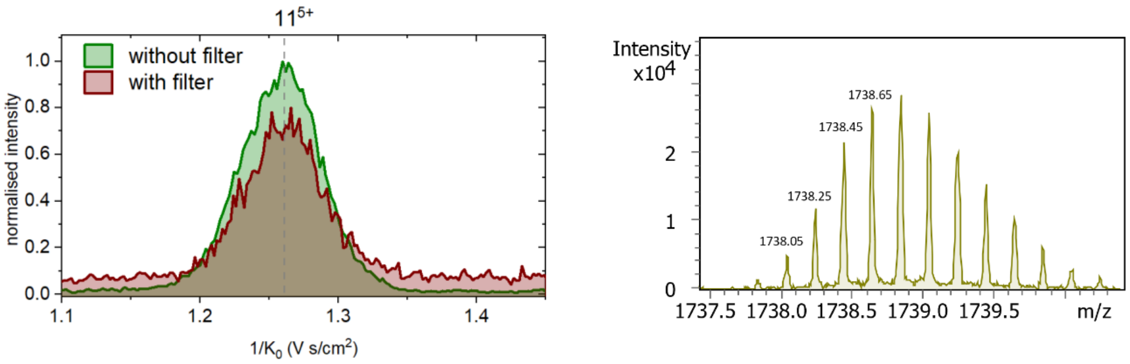

Figure P1. Extracted ion mobility spectra of *m/z* 1738 (11^{5+}) without quadrupole filter (green) and with quadrupole transmitting only selected *m/z* (red). The right panel shows the extracted mass spectrum from the mobility peak without quadrupole filter.

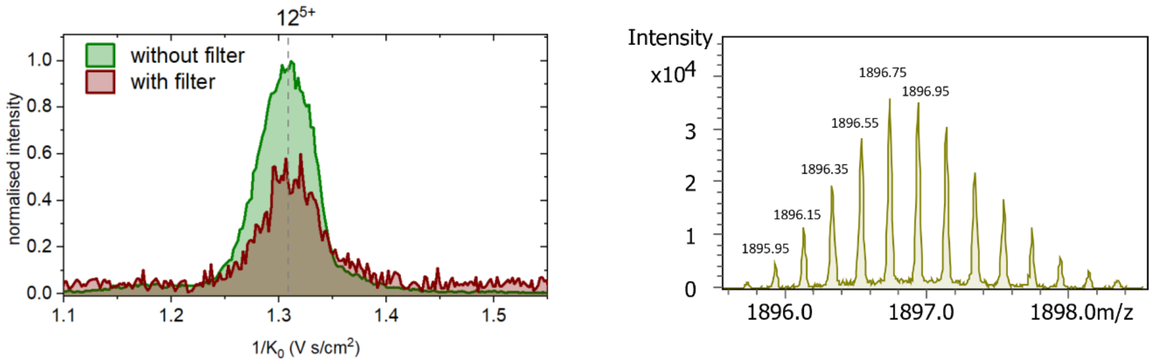

Figure P2. Extracted ion mobility spectra of *m/z* 1896 (12^{5+}) without quadrupole filter (green) and with quadrupole transmitting only selected *m/z* (red). The right panel shows the extracted mass spectrum from the mobility peak without quadrupole filter.

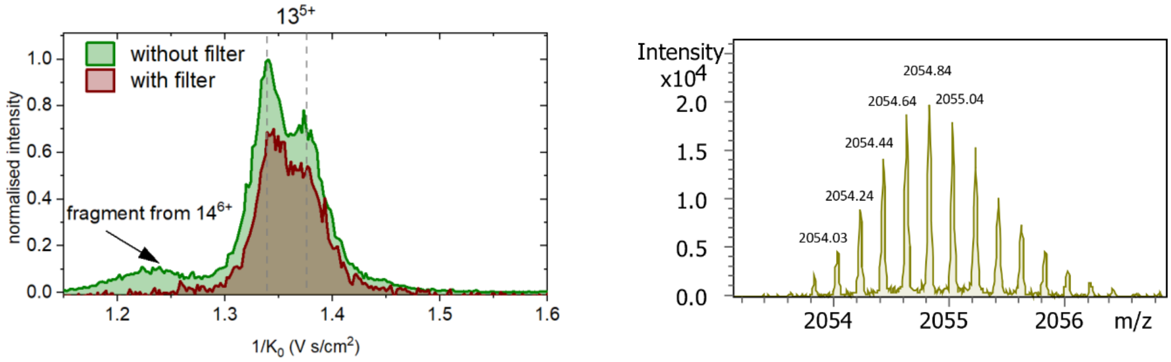

Figure P3. Extracted ion mobility spectra of *m/z* 2054 (13^{5+}) without quadrupole filter (green) and with quadrupole transmitting only selected *m/z* (red). This oligomer has two conformations at $1/K_0$=1.34 and 1.38 V·s/cm^2 that are highlighted with grey dashed lines. The right panel shows the extracted mass spectrum from the mobility peaks without quadrupole filter.

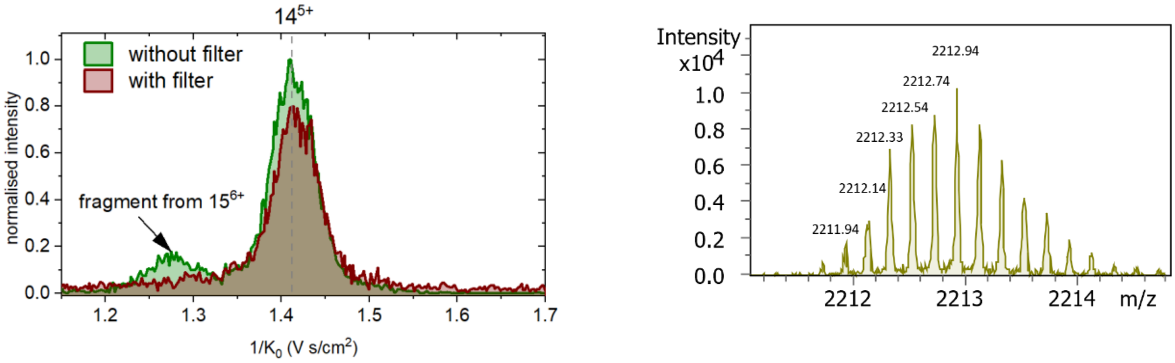

Figure P4. Extracted ion mobility spectra of *m/z* 2212 (14^{5+}) without quadrupole filter (green) and with quadrupole transmitting only selected *m/z* (red). The right panel shows the extracted mass spectrum from the mobility peak without quadrupole filter.

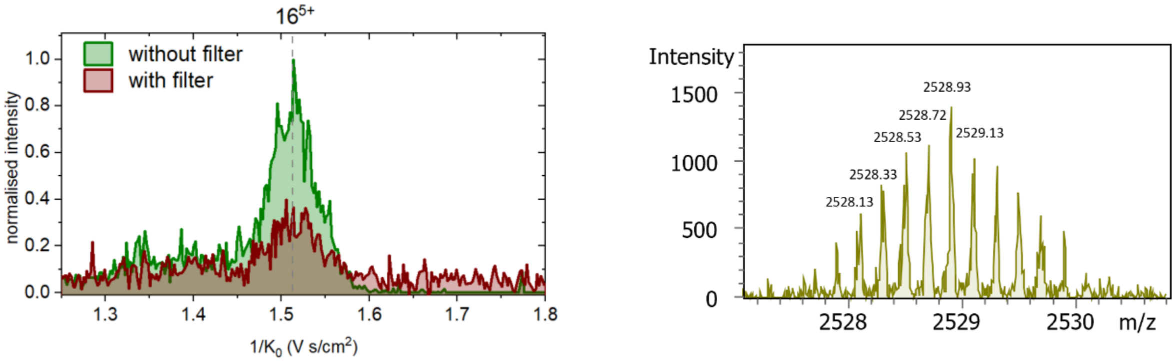

Figure P5. Extracted ion mobility spectra of *m/z* 2527 (16^{5+}) without quadrupole filter (green) and with quadrupole transmitting only selected *m/z* (red). The right panel shows the extracted mass spectrum from the mobility peak without quadrupole filter.

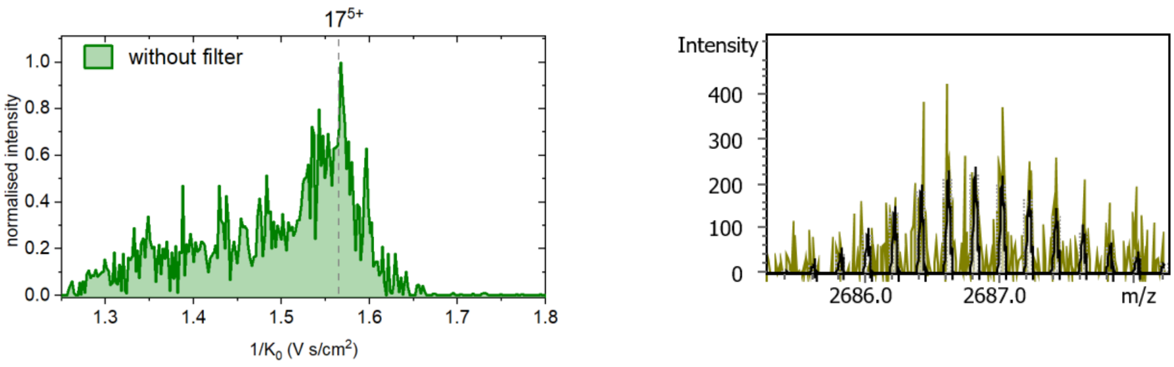

Figure P6. Extracted ion mobility spectrum of *m/z* 2685 (17^{5+}) without quadrupole filter (green). Low abundance of this oligomers did not give sufficient signal for the quadrupole selection. The right panel shows the extracted mass spectrum from the mobility peak without quadrupole filter. The black trace here corresponds to the theoretical isotopic distribution.

5. Overview of the 6+ charged oligomers

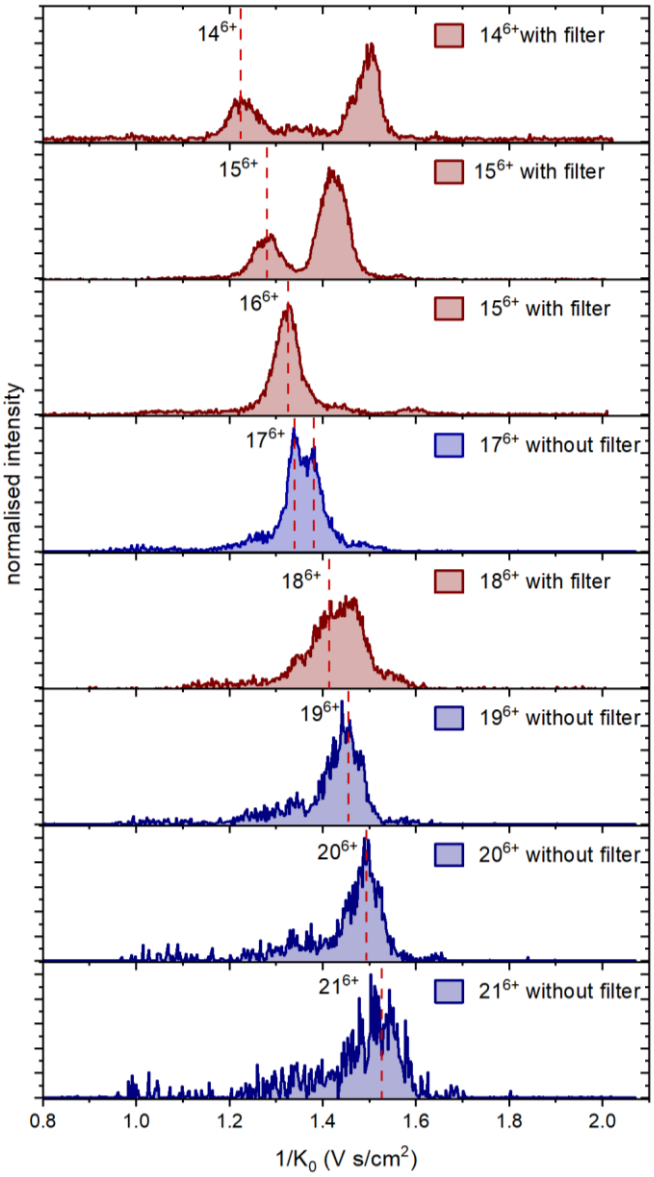

Figure H1. Extracted ion mobility spectra of the oligomers with a 6+ charge state. The quadrupole filtered ion mobility spectra are shown in red, while the blue traces correspond to the extracted ion mobilograms of the *m/z* peaks that were measured without quadrupole selection. The dashed red lines correspond to the peaks that belong to the 6+ oligomers.

References

(1) Ruotolo, B. T.; Benesch, J. L. P.; Sandercock, A. M.; Hyung, S.-J.; Robinson, C. V. Ion Mobility–Mass Spectrometry Analysis of Large Protein Complexes. *Nat Protoc* **2008**, *3*, 1139–1152. https://doi.org/10.1038/nprot.2008.78.
(2) Bush, M. F.; Hall, Z.; Giles, K.; Hoyes, J.; Robinson, C. V.; Ruotolo, B. T. Collision Cross Sections of Proteins and Their Complexes: A Calibration Framework and Database for Gas-Phase Structural Biology. *Anal. Chem.* **2010**, *82*, 9557–9565. https://doi.org/10.1021/ac1022953.
(3) Smith, D.; Knapman, T. W.; Campuzano, I.; Malham, R. W.; Berryman, J. T.; Radford, S. E.; Ashcroft, A. E. Deciphering Drift Time Measurements from Travelling Wave Ion Mobility Spectrometry-Mass Spectrometry Studies. *European Journal of Mass Spectrometry* **2009**, *15*, 113–130. https://doi.org/10.1255/ejms.947.
(4) Scarff, C. A.; Thalassinos, K.; Hilton, G. R.; Scrivens, J. H. Travelling Wave Ion Mobility Mass Spectrometry Studies of Protein Structure: Biological Significance and Comparison with X-Ray Crystallography and Nuclear Magnetic Resonance Spectroscopy Measurements. *Rapid Communications in Mass Spectrometry* **2008**, *22*, 3297–3304. https://doi.org/10.1002/rcm.3737.
(5) Hofmann, J.; Struwe, W. B.; Scarff, C. A.; Scrivens, J. H.; Harvey, D. J.; Pagel, K. Estimating Collision Cross Sections of Negatively Charged N-Glycans Using Traveling Wave Ion Mobility-Mass Spectrometry. *Anal. Chem.* **2014**, *86*, 10789–10795. https://doi.org/10.1021/ac5028353.
(6) Pagel, K.; Harvey, D. J. Ion Mobility–Mass Spectrometry of Complex Carbohydrates: Collision Cross Sections of Sodiated N-Linked Glycans. *Anal. Chem.* **2013**, *85*, 5138–5145. https://doi.org/10.1021/ac400403d.
(7) Richardson, K.; Langridge, D.; Dixit, S. M.; Ruotolo, B. T. An Improved Calibration Approach for Traveling Wave Ion Mobility Spectrometry: Robust, High-Precision Collision Cross Sections. *Anal. Chem.* **2021**, *93*, 3542–3550. https://doi.org/10.1021/acs.analchem.0c04948.
(8) Duez, Q.; Chirot, F.; Liénard, R.; Josse, T.; Choi, C.; Coulembier, O.; Dugourd, P.; Cornil, J.; Gerbaux, P.; De Winter, J. Polymers for Traveling Wave Ion Mobility Spectrometry Calibration. *J. Am. Soc. Mass Spectrom.* **2017**, *28*, 2483–2491. https://doi.org/10.1007/s13361-017-1762-4.
(9) Gelb, A. S.; Jarratt, R. E.; Huang, Y.; Dodds, E. D. A Study of Calibrant Selection in Measurement of Carbohydrate and Peptide Ion-Neutral Collision Cross Sections by Traveling Wave Ion Mobility Spectrometry. *Anal. Chem* **2014**. https://doi.org/10.1021/ac503379e.
(10) Pang, X.; Jia, C.; Chen, Z.; Li, L. Structural Characterization of Monomers and Oligomers of D-Amino Acid-Containing Peptides Using T-Wave Ion Mobility Mass Spectrometry. *J. Am. Soc. Mass Spectrom.* **2017**, *28*, 110–118. https://doi.org/10.1007/s13361-016-1523-9.
(11) Stow, S. M.; Causon, T. J.; Zheng, X.; Kurulugama, R. T.; Mairinger, T.; May, J. C.; Rennie, E. E.; Baker, E. S.; Smith, R. D.; McLean, J. A.; Hann, S.; Fjeldsted, J. C. An Interlaboratory Evaluation of Drift Tube Ion Mobility−Mass Spectrometry Collision Cross Section Measurements. **2017**. https://doi.org/10.1021/acs.analchem.7b01729.
(12) Kwon, J.; Lee, M.; Na, S. Sodium Chloride's Effect on Self-Assembly of Diphenylalanine Bilayer. *Journal of Computational Chemistry* **2016**, *37*, 1839–1846. https://doi.org/10.1002/jcc.24404.
(13) Arya, S.; Ganguly, P.; Arsiccio, A.; Claud, S. L.; Trapp, B.; Schonfeld, G. E.; Liu, X.; Cantrell, K. L.; Shea, J.-E.; Bowers, M. T. Terminal Capping of an Amyloidogenic Tau Fragment Modulates Its Fibrillation Propensity. *J. Phys. Chem B* **2020**, *124*, 8772–8783. https://doi.org/10.1021/acs.jpcb.0c05768.
